# Dissolving Microneedle for Maintaining the Integrity of HPV Virus‐Like Particles Enabling Durable Sterile Protection Across Various Mucosal Tissues

**DOI:** 10.1002/adhm.202500963

**Published:** 2025-07-09

**Authors:** Hyemi Kim, Inhyuk Hwang, Juhee Seo, Chaiwon Kim, In‐Jeong Choi, Miran Kang, Min‐Seok Rha, Youngjae Hong, Jooyoung Kim, Seung‐Ki Baek, Jung‐Hwan Park, Hyung‐Ju Cho, Kihyuck Kwak

**Affiliations:** ^1^ Department of Microbiology and Immunology Yonsei University College of Medicine 50–1 Yonsei‐ro, Seodaemun‐gu Seoul 03722 Republic of Korea; ^2^ Brain Korea 21 Project for Medical Science Yonsei University College of Medicine 50–1 Yonsei‐ro, Seodaemun‐gu Seoul 03722 Republic of Korea; ^3^ Department of Otorhinolaryngology Yonsei University College of Medicine 50–1 Yonsei‐ro, Seodaemun‐gu Seoul 03722 Republic of Korea; ^4^ QuadMedicine R&D Centre QuadMedicine, Inc. sagimakgol‐ro, 45beon‐gil, jungwon‐gu seongnam‐si Gyeonggi‐do 13209 Republic of Korea; ^5^ The Airway Mucus Institute Yonsei University College of Medicine 50–1 Yonsei‐ro, Seodaemun‐gu Seoul 03722 Republic of Korea; ^6^ Department of BioNano Technology Gachon University 1342, Seongnam‐daero, Sujeong‐gu Seongnam‐si Gyeonggi‐do 13120 Republic of Korea

**Keywords:** buccal vaccination, dissolving microneedle, human papilloma virus, long‐term immune response, mucosal immunity, neutralizing antibodies

## Abstract

Human papillomavirus (HPV) vaccines have substantially reduced cervical cancer and other HPV‐related diseases in high‐income countries, with male vaccination addressing transmission as well as head and neck cancers. However, in low‐ and middle‐income countries, widespread vaccination efforts are hindered by considerable costs, insufficient medical staff, and challenges in maintaining vaccine stability. Conventional microneedle vaccine production often compromises the structural integrity of virus‐like particles (VLPs), thereby reducing their effectiveness. To address this challenge, a dissolving microneedle platform is developed that preserves VLP stability for over six months. Administration of this vaccine platform to buccal tissue enables effective antigen delivery, inducing T follicular helper (Tfh) cell differentiation, germinal center formation, and the production of high‐titer neutralizing antibodies. Buccal application also conferred sterilizing immunity in both buccal and vaginal tissues. Moreover, the vaccine induced durable immune memory, with memory B cells and plasma cells persisting in buccal tissue and bone marrow for six months. This innovation addresses critical barriers in resource‐limited regions by enhancing VLP stability and accessibility. Furthermore, the platform's applicability extends to other vaccines requiring structural integrity for efficacy, representing a transformative approach to global immunization efforts.

## Graphical abstract

A novel dissolving microneedle platform preserves HPV virus‐like particle (VLP) immunogenicity by maintaining structural integrity, crucial for eliciting potent immune responses. Buccal administration induces robust immune response, high neutralizing antibody titers, and durable sterile protection across oral and vaginal mucosal sites. By overcoming cold‐chain limitations, this innovation significantly enhances global accessibility to effective HPV vaccination.

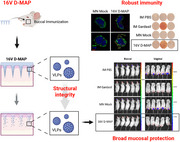

## 1 Introduction

Following the development of the HPV prophylactic vaccine in 2006, targeting HPV types 6, 11, 16, and 18, and its subsequent inclusion as a mandatory immunization in several high‐income countries, the incidence of cervical cancer and other HPV‐related diseases has declined significantly.^[^ ^1^, ^2^, ^3^ ^]^ In several countries outside the United States, HPV vaccination has also become mandatory for males, reflecting their critical role in HPV transmission and the notable incidence of HPV‐related head and neck cancers among men, though such mandates remain relatively uncommon within the U.S. itself.^[^ ^4^, ^5^ ^]^ Accumulating clinical data highlight the HPV vaccine as one of the most effective prophylactics against infection‐related cancers.^[^ ^6^ ^]^ The remarkable success of the HPV vaccine can be attributed to its virus‐like particle (VLP)‐based platform.^[^ ^7^, ^8^ ^]^ Such VLP‐based vaccines are highly effective because they repetitively arrange the epitopes recognized by B cells on a single particle, thereby enabling robust B cell receptor (BCR) cross‐linking.^[^ ^9^ ^]^ This robust cross‐linking results in strong signaling pathways and amplifies the B cell response and humoral immunity, often by tens to hundreds of fold.^[^ ^10^, ^11^ ^]^ The formation and stability of this highly immunogenic VLP platform are essential to the HPV vaccine's efficacy, underscoring the critical role of VLPs in its success.^[^ ^12^, ^13^ ^]^

To preserve the integrity and stability of VLPs, the key drivers of HPV vaccine efficacy, a cold chain (+2 °C to +8 °C) is required during transportation, along with refrigeration at the destination.^[^ ^14^ ^]^ This cold‐storage requirement substantially increases costs, posing a significant barrier to distribution, especially in low‐ and middle‐income countries (LMICs), where the incidence of HPV‐related cancers is elevated because of limited pap‐smear screening programs.^[^ ^15^, ^16^ ^]^ Notably, nearly all cases of cervical cancer are driven by HPV infection, and the burden disproportionately affects low‐income countries, where age‐standardized incidence rates reach 9.2 cases per 100,000 compared to 6.9 cases per 100,000 in high‐ and upper‐middle‐income regions.^[^ ^17^ ^]^ Consequently, developing an HPV vaccine that eliminates cold‐chain logistics is crucial for improving accessibility and lowering the global burden of HPV‐related diseases.^[^ ^18^ ^]^ Beyond cold chain obstacles, the limited availability of medical personnel and inadequate healthcare infrastructure further restrict HPV vaccine distribution and administration in LMICs.^[^ ^19^ ^]^ Consequently, the creation of a simplified delivery platform that does not require trained medical staff, coupled with the capacity to store and transport the vaccine at ambient temperatures, is recognized as a vital strategy to enhance vaccine accessibility and uptake in resource‐constrained settings.^[^ ^20^ ^]^

Microneedles, microscopic projections typically 200–700 micrometers in length, have recently emerged as a promising platform for antigen delivery, addressing several longstanding obstacles related to vaccine distribution, storage, and administration.^[^ ^21^ ^]^ The Vaccine Innovation Prioritization Strategy (VIPS) Alliance, a collaboration among Gavi, the Bill & Melinda Gates Foundation, UNICEF, PATH, and WHO, has acknowledged microneedle platform technology as an innovative delivery device that enhances equitable vaccine access and strengthens global health security, particularly in LMICs.^[^ ^22^ ^]^ Notably, microneedle‐based delivery systems, including dissolving and solid‐coated microarray patches (MAPs), are prioritized to meet the target product profile requirements for HPV vaccines.^[^ ^23^ ^]^ Among the 11 priority vaccine targets for MAPs identified in the VIPS Alliance action plan, HPV vaccines are classified under Priority Group 1 due to their strong potential for financial sustainability, high funder interest, and significant programmatic impact when delivered via MAP format.^[^ ^24^ ^]^

Dissolving MAPs (D‐MAPs) are fabricated by drying a blend of biocompatible, needle‐forming materials and antigens, which enables vaccine stability at ambient temperatures and eliminates the need for cold‐chain requirements.^[^ ^25^ ^]^ These patches can be applied directly to the skin or mucosal surfaces using short, dissolvable needles, removing the requirement for traditional injection‐based delivery methods.^[^ ^26^ ^]^ This innovation facilitates widespread vaccination without requiring extensive medical infrastructure or specialized personnel. However, conventional microneedle fabrication methods face major challenges in preserving the structural integrity of complex antigens, including HPV VLPs composed of 360 L1 proteins.^[^ ^27^ ^]^ During production, VLPs are exposed to destabilizing stressors, including mechanical forces, temperature fluctuations, solvent exposure, and dehydration.^[^ ^28^ ^]^ These factors can compromise the VLPs' structural conformation, leading to degradation, a reduction in antigen multivalency, and ultimately, a loss of immunogenicity. Maintaining VLP stability throughout D‐MAP production is therefore crucial to preserving microneedle‐based HPV vaccine efficacy.

In this study, we developed an optimized D‐MAP manufacturing process that preserves the structural integrity of HPV VLPs post‐dissolution. Using this process, HPV VLPs in D‐MAPs remained structurally intact for 7 days under accelerated storage and for up to 6 months at room temperature, as verified by protein electrophoresis, enzyme‐linked immunosorbent assay (ELISA), transmission electron microscopy (TEM), and dynamic light scattering (DLS). In a mouse model, D‐MAPs administered via the buccal mucosa effectively delivered HPV VLPs to draining lymph nodes, eliciting a robust humoral immune response characterized by the induction of antigen‐specific B cells. This efficient antigen delivery through the buccal mucosa conferred antibody‐mediated immunity against HPV infections, providing cross‐protection in both buccal and vaginal tissues. The immune response exhibited long‐term persistence, with memory B cells and plasma cells detected in these tissues. Moreover, protective immunity was sustained in in vivo buccal and vaginal challenge models up to six months post‐immunization. By incorporating VLPs into dissolving microneedles in a stable manner, our study presents a cost‐effective and efficient vaccine platform capable of preventing HPV‐related genital and head and neck cancers through buccal administration alone. Further, this D‐MAP platform holds significant potential for application to other VLP‐based vaccines with structurally vulnerable antigens, offering an accessible and affordable vaccination solution for LMICs.

## 2 Results and Discussion

### 2.1 Production and Characterization of the HPV16 VLP D‐MAP

**Figure 1:**
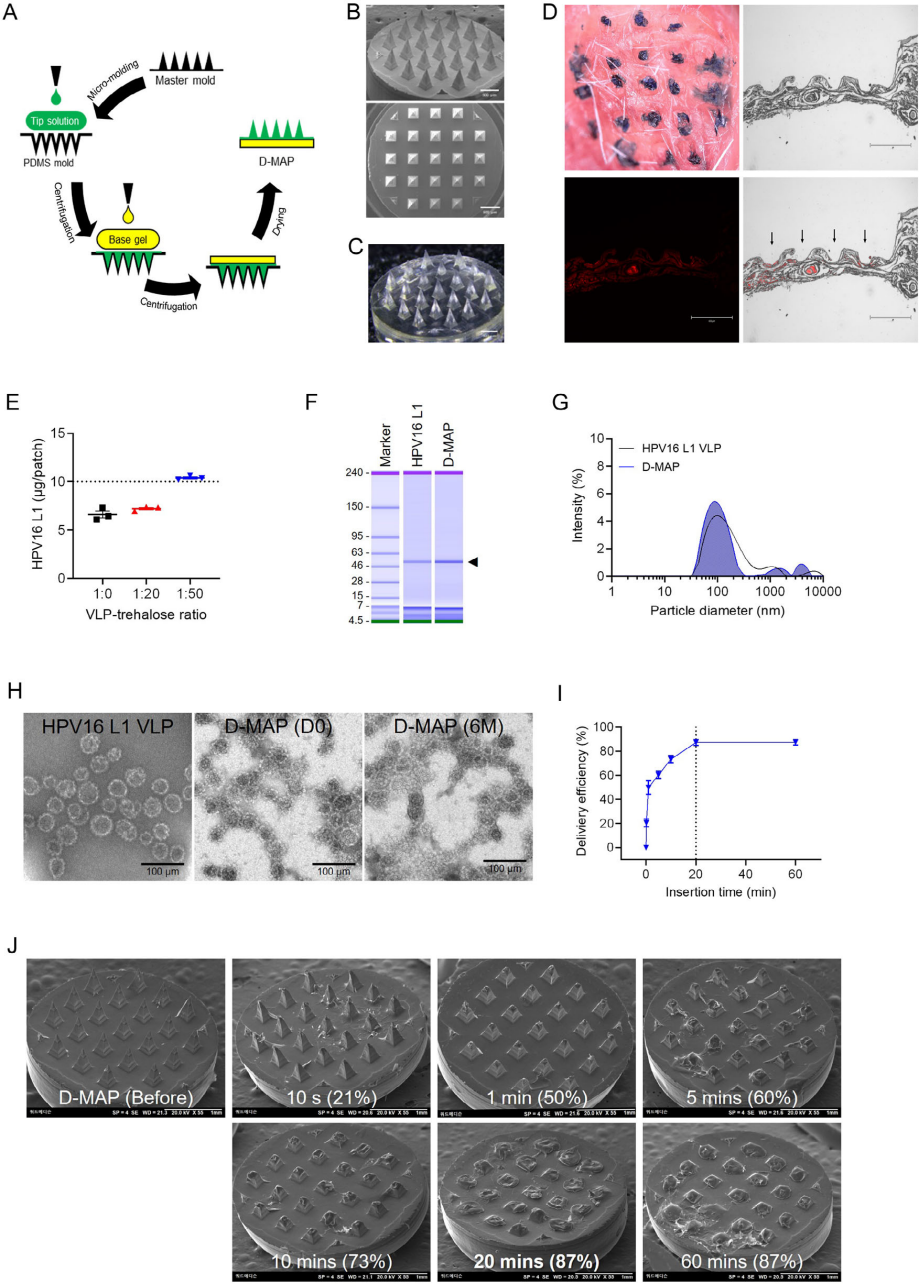
Fabrication and characterization of an HPV virus‐like particle (VLP) D‐MAP using centrifugal casting. A) Schematic diagram of the centrifugal casting process for HPV D‐MAP. B) Scanning electron microscopy (SEM) image of the microneedle morphology. C) Bright field microscopic image of HPV D‐MAP. D) Ex vivo evaluation of insertion and delivery performance of 16 V D‐MAP on mouse buccal mucosa. (Top left) Bright‐field microscopy image illustrating microchannels created by trypan blue‐loaded microneedles. (Top right) Bright‐field microscopy image following the application of rhodamine B‐loaded microneedles. (Bottom left) Fluorescence microscopy image demonstrating localized fluorescence at the insertion sites. (Bottom right) Merged bright‐field and fluorescence microscopy image confirming precise microneedle placement and delivery. Microneedles were applied for 20 min prior to tissue section preparation and imaging. E) Antigen content in HPV D‐MAP with ratios of 1:0, 1:20, and 1:50 of VLP to trehalose. F) Comparative analysis of HPV16 L1 VLP by protein electrophoresis, before and after HPV D‐MAP production. G) Dynamic light scattering (DLS) analysis demonstrating particle size distribution. H) Transmission electron microscopy (TEM) image confirming the structural integrity of VLPs encapsulated within 16V D‐MAP. I) Delivery efficiency of 16 V D‐MAP at various application intervals. J) Scanning electron microscopy (SEM) images illustrating the structural changes of microneedles before insertion and after insertion into mouse buccal mucosa at specific time intervals (10 s, 1, 5, 10, 20, and 60 min).

To maximize the immunogenicity of HPV16 VLPs, the development of a D‐MAP platform requires careful consideration of key factors: the formation of microtrauma upon tissue application, the preservation of VLP integrity during microneedle fabrication, and after microneedle dissolution. We have successfully identified and optimized fabrication conditions that satisfy these critical requirements, ensuring both effective antigen delivery and robust immunogenic response. The characteristics and mechanical performance of HPV16 VLP D‐MAP (16 V D‐MAP) manufactured by micro‐molding technology (**Figure** **1**A,B) were evaluated. The 16 V D‐MAP, composed of 21 tips, was confirmed to have no bending or breaking at the tip ends when observed through an optical microscope (Figure 1C). In the insertion test on porcine skin, 100% penetration efficiency was observed upon staining the puncture holes caused by the 21 tips of the 16 V D‐MAP with trypan blue (Figure S1A, Supporting Information). Given that the extracellular matrix of the buccal mucosa possesses a looser architecture and a higher elastin content compared to skin,^[^ ^29^ ^]^ we further evaluated the insertion efficiency and dissolution characteristics of microneedles using *ex vivo* mouse buccal mucosa. The 16 V D‐MAP was manually applied onto the buccal mucosa for a duration of 20 min, which provided sufficient time for effective needle dissolution. Upon application of the trypan blue‐loaded 16 V D‐MAP, clear visualization of the dye was observed in the tissue (Figure 1D, upper left). To further confirm the transfer of the dissolved needle components into the tissue, 16 V D‐MAPs containing rhodamine B were applied to the *ex vivo* buccal mucosa. Examination of longitudinal sections revealed localized fluorescence at the needle insertion sites, demonstrating successful payload delivery (Figure 1D, lower left). The merged imaging clearly indicated accurate microneedle placement and effective dissolution (Figure 1D, lower right). Collectively, these findings illustrate that the 16 V D‐MAP exhibits adequate mechanical integrity and efficient payload transfer capability, making it well‐suited for application in soft mucosal tissues and suitable for subsequent in vivo evaluations.

In order to determine an optimal formulation that maintains the antigen content during manufacturing, 16 V D‐MAPs containing varying amounts of trehalose were produced and dissolved in phosphate‐buffered saline (PBS) for characterizing the loaded VLP extracts. Among the 16 V D‐MAP formulations set at a ratio of 1:0, 1:20, and 1:50 of VLP to trehalose, the antigen content of 16 V D‐MAP only reached the expected amount of 10 µg at a ratio of 1:50 (1:0, 6.62 ± 0.64 µg patch^−1^; 1:20, 7.22 ± 0.23 µg patch^−1^; 1:50, 10.41 ± 0.22 µg patch^−1^) (Figure 1E). The HPV16 L1 protein, corresponding to 56 kDa, was confirmed under reducing and denaturing conditions. The purity of the intact L1 protein in liquid and microneedle formulations was calculated to be 98% and 97.9%, respectively, confirming the maintenance of integrity (Figure 1F).

The conformational epitopes and structural integrity of HPV16 L1 VLPs are known to be crucial for eliciting a protective efficacy in the host, including a neutralizing antibody response.^[^ ^30^ ^]^ Thus, it is necessary to determine the degree of aggregation and the morphological characteristics of the VLPs. In the DLS analysis, the hydrodynamic diameter of the VLP before and after 16 V D‐MAP production with a VLP to trehalose ratio of 1:50 was ≈140.7 ± 13.3 nm (polydispersity, PDI: 0.27 ± 0.02) and 121.02±4.7 nm (PDI: 0.26 ± 0.02), respectively (Figure 1G; Tables S1 and S2, Supporting Information). The structure of the VLP was observed in the TEM image as a complete, spherical particle in both liquid and microneedle formulations. Furthermore, TEM analysis performed after 6 months of storage also confirmed the preservation of VLP morphology in the microneedle formulation, indicating long‐term structural stability (Figure 1H). Therefore, it was confirmed that HPV16 L1 VLP showed no aggregation and was highly homogeneous in the microneedle formulation.

To determine the optimal attachment time required for effective antigen delivery using the 16 V D‐MAP, we initially conducted an *ex vivo* penetration and dissolution test using porcine skin at several intervals (0, 10 s, 1, 5, 30, and 60 min) (Figure S1B, Supporting Information). Subsequently, to more accurately reflect the mucosal environment targeted in this study, we performed an additional in vivo penetration experiment using mouse buccal mucosa at multiple time intervals (0, 10 s, 1, 5, 10, 20, and 60 min). The antigen delivery efficiency, calculated based on the residual HPV16 L1 antigen content remaining in the microneedle tips of 16 V D‐MAP, increased rapidly within the first minute (50%), and plateaued at ≈87% by 20 min post‐administration (Figure 1I). Therefore, we selected 20 min as the optimal attachment time for the 16 V D‐MAP on mouse buccal mucosa for subsequent experiments.

### 2.2 Features of 16 V D‐MAP Formulation to Maintain Stability

At all stages of VLP‐based vaccine manufacturing and storage, it is crucial to preserve the native conformation of protective epitopes within the VLPs. Developing an appropriate formulation is essential to stabilize VLPs against chemical and physical stresses. Trehalose, a pharmaceutical excipient, stabilizes proteins both in liquid form by preferential hydration and in solid form through interactions that form viscous glassy matrices. The stability of proteins increases with higher trehalose ratios.^[^ ^31^ ^]^

**Figure 2:**
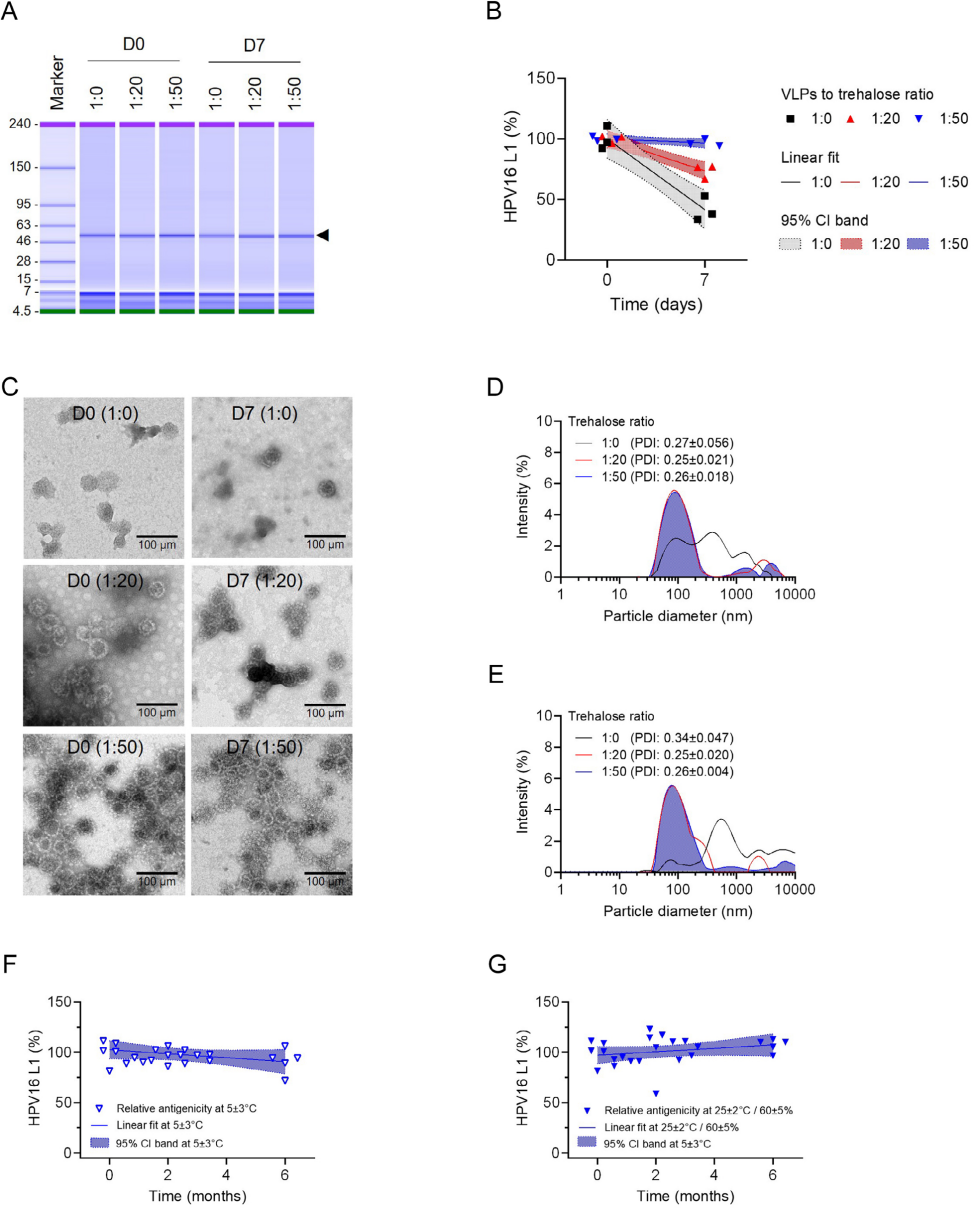
Preformulation assessments for optimal stabilizer composition and long‐term stability evaluation in HPV D‐MAP. The characteristics of HPV16 L1 VLP were evaluated in HPV D‐MAP formulations with ratios of 1:0, 1:20, and 1:50 of VLP to trehalose using A) protein electrophoresis, B) ELISA, C) TEM, and D,E) DLS analysis before and after 7 days under accelerated storage conditions (40 ± 2 °C/75 ± 5% RH). These experiments were conducted with n = 3 per group (A–E). The DLS graphs (D–E) represent mean values including hydrodynamic diameter and PDI (mean±SD). The long‐term stability of HPV D‐MAP, formulated with a 1:50 ratio of VLP to trehalose, was assessed under F) refrigeration (5 ± 3 °C) and G) room temperature (25 ± 2 °C / 60 ± 5% RH), with n = 5 per group (F–G).

In this study, we confirmed the trehalose formulation that maintains the quality and stability of 16 V D‐MAP, along with the carboxymethylcellulose (CMC) compositions, ensuring the mechanical strength of microneedles. The purity of the L1 protein in the 16 V D‐MAP formulations, formulated at ratios of 1:0, 1:20, and 1:50 of VLP to trehalose, was consistently measured at 98%. After 7 days under accelerated storage conditions (40 ± 2 °C / 75 ± 5% relative humidity [RH]), no significant differences were observed among all formulations, indicating that the integrity of the intact L1 protein remains unaffected by both trehalose concentration and storage conditions (**Figure** **2**A). Specifically, only the 16 V D‐MAP formulation with a 1:50 ratio of VLP to trehalose preserved antigen content over the 7‐day accelerated storage period (Slope value: −0.49). In contrast, 16 V D‐MAP formulations without a stabilizer (Slope value: −8.36) or with a 20‐fold of trehalose (Slope value: −3.77) showed a rapid decrease in antigenicity under accelerated conditions (Figure 2B).

TEM and DLS analyses confirmed that there were no morphological changes or aggregation of VLPs in 16 V D‐MAP formulations manufactured with a VLP to trehalose ratio of 1:50 over the 7‐day accelerated condition (Figure 2C,E). However, TEM images indicated that the spherical structure of VLPs decreased and aggregated when formulated with less than a 20‐fold excess of trehalose over the same period (Figure 2C). In DLS analysis, the hydrodynamic diameter of VLPs before and after 7 days under accelerated storage conditions with a VLP to trehalose ratio of 1:50 was ≈121.02 ± 4.7 nm (PDI: 0.26 ± 0.02) and 113.2 ± 6.59 nm (PDI: 0.26 ± 0.004), respectively. As the ratio of VLPs to trehalose within the 16 V D‐MAP formulation decreased by less than 20‐fold, the size distribution of VLPs became broader and polydispersity increased under accelerated conditions (Figure 2D,E; Tables S2–S7, Supporting information). These results indicate the necessity of trehalose formulations exceeding a 50‐fold excess to maintain the quality and stability of 16 V D‐MAP.

We have confirmed the long‐term stability of 16 V D‐MAP under refrigerated (5 ± 3 °C) and room temperature (25 ± 2 °C / 60 ± 5% RH) conditions for 6 months. The antigenicity of 16 V D‐MAP, formulated with a 1:50 ratio of VLP to trehalose, remains stable during storage at both temperatures. Importantly, this stability is expected to persist for up to 6 months, as indicated by stable slope values under both refrigerated (Slope value: −1.96) and room temperature (Slope value: 1.7) storage conditions (Figure 2F,G).

### 2.3 Buccal 16 V D‐MAP Administration Enables Efficient Delivery of HPV16 VLP Antigens to Antigen‐Specific B Cells

For 16 V D‐MAP vaccine to be effective, the microneedles applied to the buccal tissue must dissolve, allowing the release and delivery of the antigen to the target tissue. The antigens have to be subsequently transported to the draining lymph nodes, where it is ultimately captured by antigen‐specific B cells via their B cell receptors (BCRs). To track the effective delivery of VLP antigens to the immune system, we chemically conjugated Alexa Fluor 647 on HPV16 VLPs surface and loaded them into the microneedles (AF647 16 V D‐MAP). These microneedles were then applied to the buccal tissue of mice. At 1 h post‐application, the delivery of VLPs to the buccal tissue was analyzed, while at 24 h post‐application, the draining lymph nodes and antigen‐specific B cells were assessed using the i(n vivo imaging system (IVIS) and flow cytometry, respectively.

**Figure 3:**
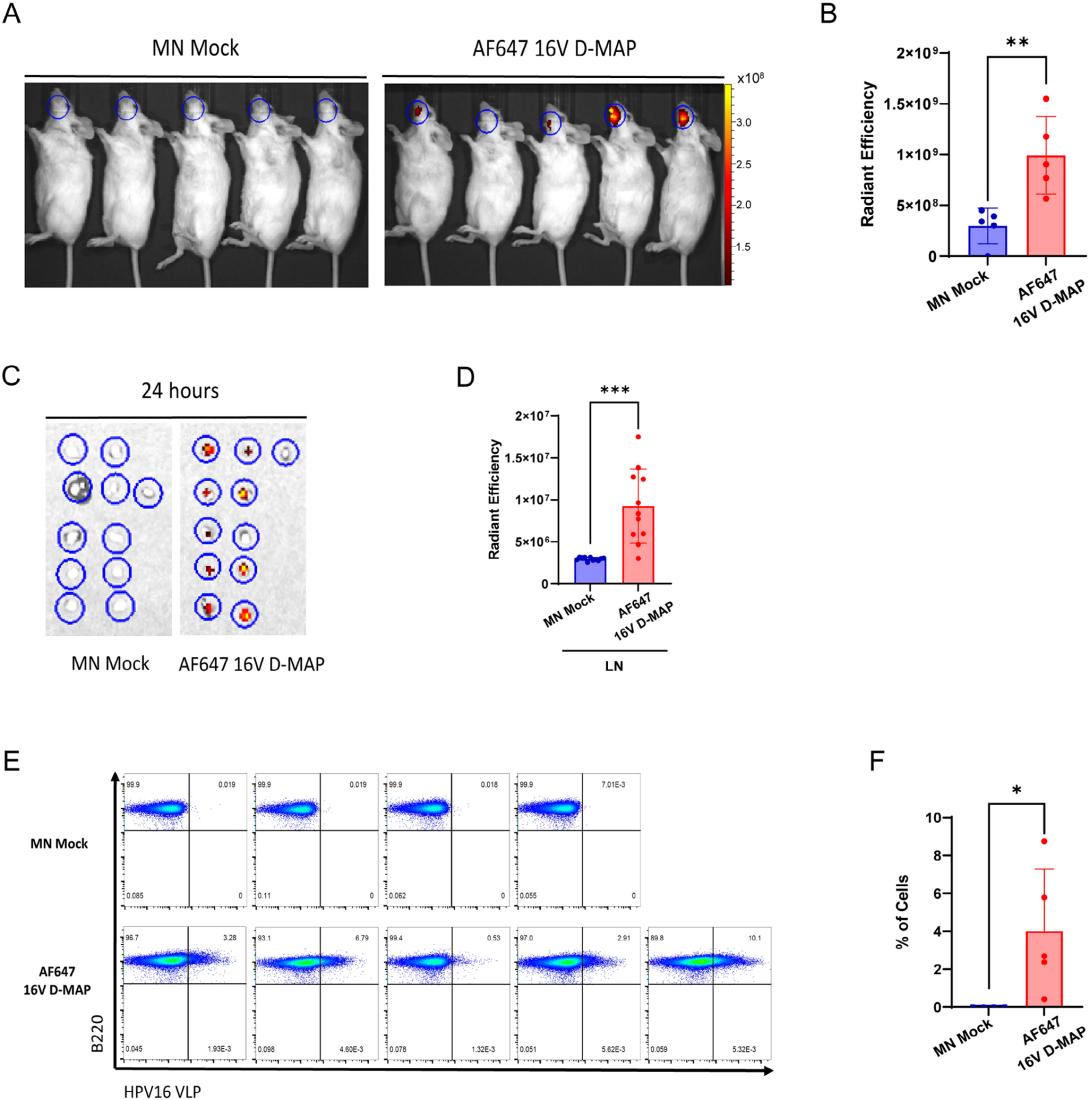
Delivery of HPV16 VLPs to immune cells via microneedles through buccal tissue. A) Alexa Fluor 647 conjugated HPV16 VLP microneedles (AF647 16 V D‐MAP) were administered to the buccal mucosa of mice for 20 min. Bioluminescence imaging was performed 1 h post‐administration (n = 5 per group). Radiant efficiency was measured in units of (p/s/cm^2^/sr)/(µW/cm^2^), and the color scale ranged from 1.03 × 10⁸ to 3.45 × 10⁸. B) Quantitative analysis of fluorescence imaging data from the buccal areas of MN Mock and AF647 16 V D‐MAP treated mice. C) Fluorescence imaging of cervical lymph nodes, and D) quantification of antigen delivery to cervical lymph nodes 24 h after microneedle administration. E) Flow cytometric analysis of HPV16 VLP‐specific B cells in the cervical lymph nodes. F) Statistical analysis of HPV16 VLP‐specific cells in cervical lymph nodes. Data are presented as means ± SEM. ns (not significant),p > 0.05; ^*^ *p* < 0.05; ^**^ *p* ≤ 0.01; ^****^ *p* ≤ 0.0001 (unpaired *t*‐test).

After applying the microneedles to the buccal tissue, they were removed, and the IVIS imaging system was used to evaluate the absorption and retention of the dissolved HPV16 VLP antigens within the buccal tissue, ensuring no leakage back into the oral cavity. A strong signal detected from the buccal tissue confirmed the successful dissolution and localized delivery of the antigens (**Figure** **3**A,B). At 24 h post‐immunization, to assess whether the antigens had been further transported to the draining lymph nodes, the cervical lymph nodes were harvested, and the amount of antigen delivered to the lymph nodes was quantified via fluorescent signal analysis. Robust signals in the lymph nodes confirmed that the antigens, initially delivered to the buccal tissue, were effectively absorbed and transported to the draining lymph nodes (Figure 3C,D). For a successful humoral immune response, antigen presentation to antigen‐specific B cells via BCRs in the lymph nodes is essential. To confirm this, B cells were purified from the harvested lymph nodes, and flow cytometry was conducted to detect AF647 signals, indicating that HPV16 VLPs had been delivered to antigen‐specific B cells. No signal was detected in the B cells of the control group treated with mock microneedles. In contrast, fluorescence was observed in an average of 4% of B cells, including those considered to be antigen‐specific, in the group treated with AF647‐labeled 16 V D‐MAP‐loaded microneedles (Figure 3E,F), indicating the successful uptake of HPV16 VLP antigens by cognate B cells. These results demonstrate that the 16 V D‐MAP microneedles effectively delivered antigens sequentially to the buccal tissue, draining lymph nodes, and antigen‐specific B cells, highlighting their potential for efficient immune activation.

### 2.4 16 V D‐MAP with Structurally Intact HPV16 VLP Effectively Induces a Humoral Immune Response

**Figure 4:**
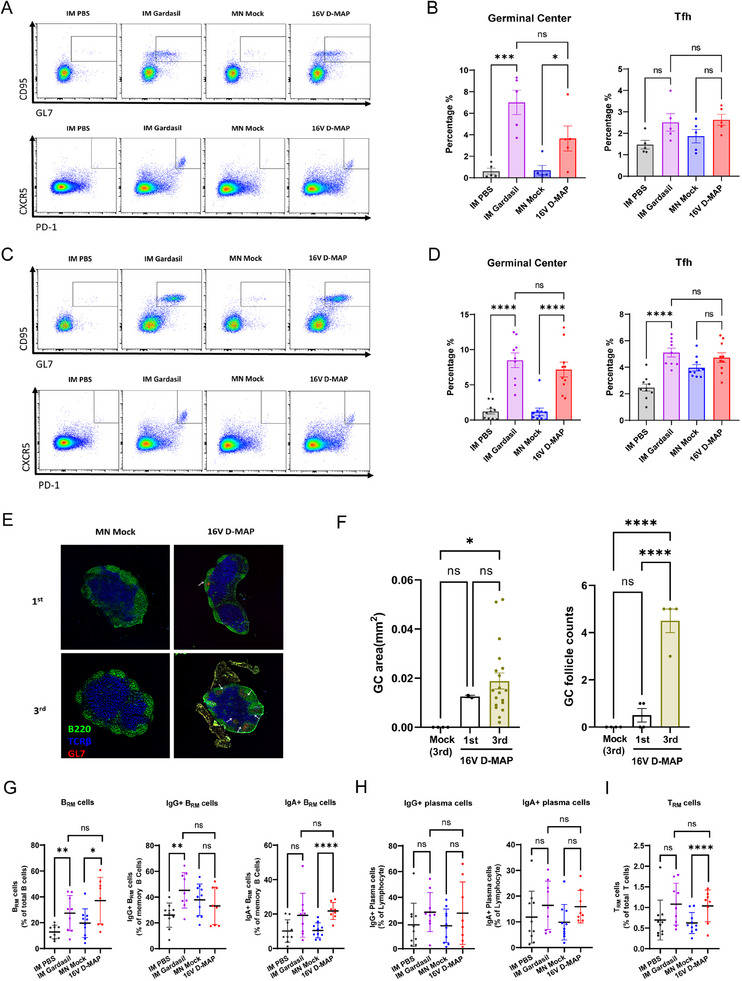
Immune responses in draining lymph nodes (LNs) following immunization with IM PBS, IM Gardasil, MN Mock, or 16 V D‐MAP. A) Representative flow cytomerty results showing GC B cell and Tfh cell populations in LNs 14 days after the first immunization with PBS, IM Gardasil, MN Mock, or 16 V D‐MAP (n = 5 per group). B) Statistical analysis of GC B cell and Tfh cell populations in LNs 14 days after the first immunization. C) Representative flow cytometry results of GC B cell and Tfh cell populations in LNs 14 days after the third immunization (n = 10 per group). D) Statistical analysis of GC B cell and Tfh cell populations in LNs 14 days after the third immunization. E) Representative confocal microscopy images of cervical LNs 14 days after the first and third immunizations with MN Mock or 16 V D‐MAP. White arrows indicate GC follicles. F) Quantification of GC follicle numbers and GC areas based on fluorescence imaging data. G–I) Statistical analysis of Brm, Trm, and plasma cells in the buccal tissue of mice following a third immunization with IM PBS, IM Gardasil, MN Mock, or 16 V D‐MAP (n = 10 per group). Data are presented as means ± SEM. ns (not significant), p > 0.05; ^*^ *p* < 0.05; ^**^ *p* ≤ 0.01; ^****^ *p* ≤ 0.0001 (one‐way ANOVA or unpaired *t*‐test).

We investigated whether 16 V D‐MAP effectively induces a strong vaccine‐mediated immune response. To evaluate the immunogenicity of HPV16 VLP antigens delivered by the 16 V D‐MAP system, mice were immunized three times at two‐week intervals (Days 0, 14, and 28). In the experimental group, antigen‐loaded dissolving microneedles were administered to the buccal mucosa. Gardasil, serving as the positive control, was delivered via intramuscular injection into the right quadriceps femoris at a dosage equivalent to one‐tenth of the standard human dose. Negative control groups included mice receiving either intramuscular (IM) injections of phosphate‐buffered saline (IM PBS) or application of mock microneedles without antigen (MN Mock) onto the buccal mucosa. Since a successful vaccine capable of inducing a high titer of neutralizing antibodies should also elicit a strong and effective germinal center (GC) response, we examined immune indicators such as the differentiation of T follicular helper (Tfh) cells and germinal center B (GC B) cells in the draining lymph nodes two weeks after the primary vaccination. Flow cytometry analysis showed that the 16 V D‐MAP group exhibited significantly higher frequency of GC B (GL7^+^CD95^+^) and Tfh (PD‐1^+^CXCR5^+^) cells compared to the negative control groups, although slightly lower than IM Gardasil, but without statistical significance (**Figure** **4**A,B). Poor vaccines typically induce a minimal GC response after a single dose, but the 16 V D‐MAP demonstrated strong immunogenicity, inducing a robust immune response. Next, we analyzed the immune response following the third vaccination by harvesting the draining lymph nodes using the same methods. After the third dose, the proportion of GC B cells exceeded 7% of the total B cell (B220^+^CD19^+^) population, and the differentiation of Tfh cells reached nearly 4% of the total CD4^+^ T cell population in 16 V D‐MAP group, indicating a strong immune response (Figure 4C,D).

To further validate the strong immunogenicity of 16 V D‐MAP observed through flow cytometry, we performed histological analysis. After the first and third vaccinations, lymph nodes from the mice were harvested to assess the extent of the GC response based on GC formation. As expected, no GC structures were observed in the lymph nodes of mice that received the mock microneedle (MN Mock) vaccination. However, in the lymph nodes of mice that received the first and third doses of 16 V D‐MAP via buccal tissue, particularly after the third vaccination, immunofluorescence staining revealed the presence of multiple GCs, marked by GL7, within the B cell follicles (Figure 4E,F). This confirmed the robust GC response induced by 16 V D‐MAP.

We further investigated whether 16 V D‐MAP vaccination induces the formation of tissue‐resident memory B (Brm) cells, tissue‐resident memory T (Trm) cells, and tissue‐resident plasma cells in the buccal tissue, which could offer rapid and effective protection against future infections (Figure S1, Supporting Information).^[^ ^32^ ^]^ Notably, we observed the formation of Brm cells and plasma cells, present at high frequencies within the tissue (Figure 4G,H). Additionally, Trm cells, which mediate cellular immunity, were effectively induced by 16 V D‐MAP vaccination (Figure 4I).

Together, these findings demonstrate that 16 V D‐MAP, by delivering structurally intact VLPs, is not only capable of eliciting a potent immune response but also facilitates the formation of memory immune cells capable of mounting a swift response to future infections. This suggests that it has the potential to efficiently promote the differentiation of plasma cells and induce high titers of neutralizing antibodies.

### 2.5 16 V D‐MAP Elicits High Levels of Antigen‐Specific Plasma Cells, Along with Neutralizing Antibodies

**Figure 5:**
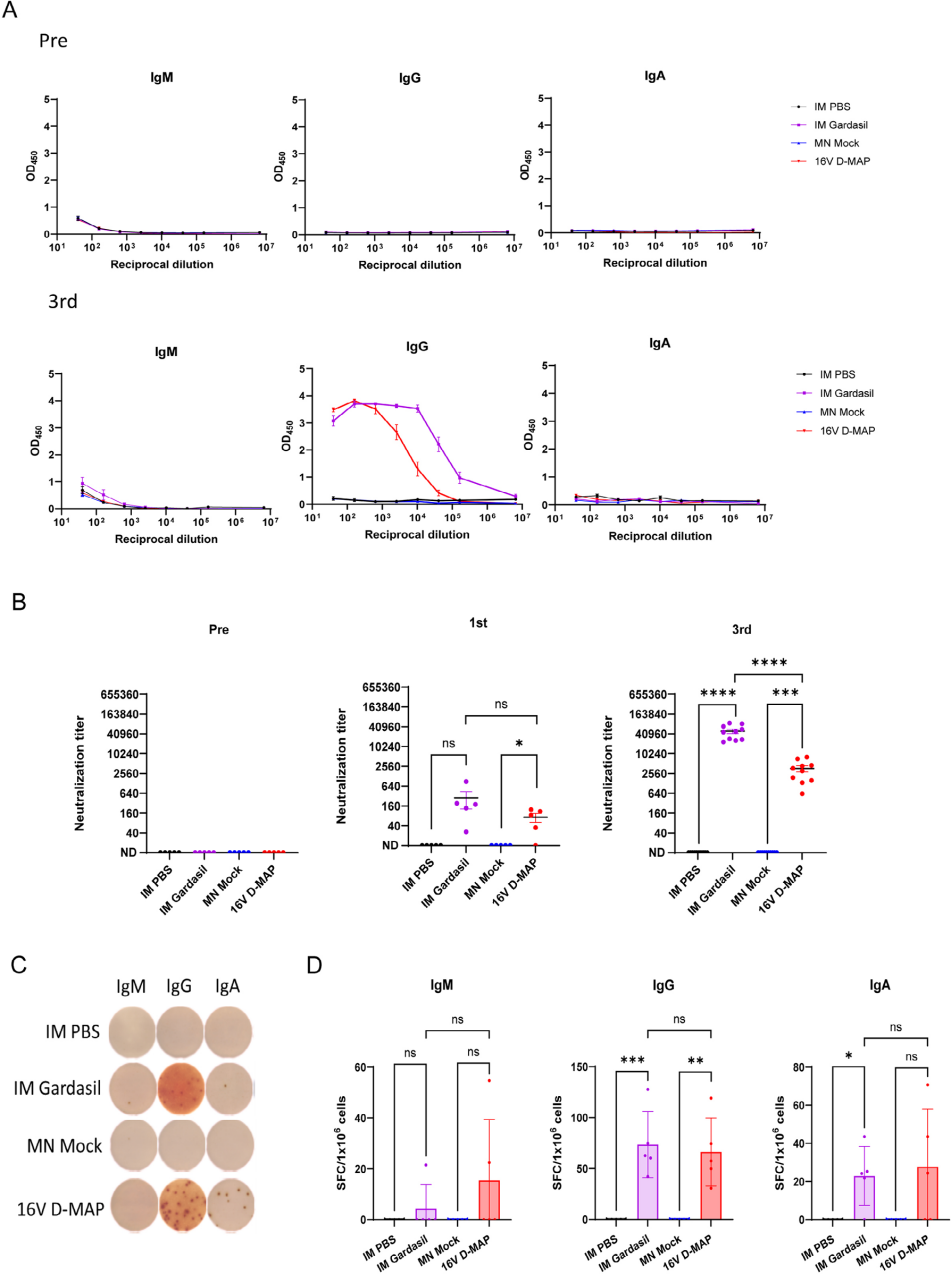
Plasma cell differentiation and neutralizing antibody responses induced by 16 V D‐MAP immunization. A) Serum levels of HPV16 VLP‐specific IgG, IgM, and IgA in IM PBS, IM Gardasil, MN Mock, and 16 V D‐MAP groups, detected by ELISA 14 days after the first and third immunization (n = 5 per group). B) Serum neutralization titers against HPV16 PsV were measured at pre‐immunization, 14 days after the first immunization (n = 5 per group), and 14 days after the third immunization (n = 10 per group). C) Enzyme‐linked immunospot (ELISPOT) assay detecting HPV16 VLP‐specific IgG‐, IgM‐, and IgA‐secreting plasma cells in bone marrow (BM) 14 days after the third immunization (n = 5 per group). D) Quantification of IgG‐, IgM‐, and IgA‐secreting plasma cells based on ELISPOT data. Data are presented as means ± SEM. Statistical significance: ns (not significant), *p* > 0.05; ^*^ *p* < 0.05; ^*^^*^ *p* ≤ 0.01; ***p ≤ 0.001; ^*^^***^ *p* ≤ 0.0001 (unpaired *t*‐test).

To further assess the antibody response as an indicator of the robust immunogenicity induced by 16 V D‐MAP, we measured the titer of antigen‐specific antibodies, the neutralizing capacity of these antibodies against HPV16 pseudoviruses (PsVs), and the quantitative presence of plasma cells in the bone marrow secreting antigen‐specific antibodies. Following the third vaccination with 16 V D‐MAP, we observed a significantly elevated titer of antigen‐specific immunoglobulin G (IgG) in the serum compared to pre‐serum samples. Although the ELISA EC50 value was approximately tenfold lower IgG titer compared to the IM Gardasil group, a substantial amount of HPV16‐specific antibodies was still elicited (**Figure** **5**A). Notably, despite the mucosal route of immunization, antigen‐specific immunoglobulin A (IgA) was undetectable in the saliva (data not shown). This absence of IgA may be due to the thin mucosal epithelium of the mouse buccal tissue, suggesting that buccal microneedle delivery in mouse model might mimic intramuscular rather than intra‐lamina propria immunization. To accurately assess the IgA‐inducing potential, experiments in animal models with thicker skin or mucosal layers are required.

Next, we evaluated the production of neutralizing antibodies against HPV16 two weeks after both the first and third vaccinations. 16 V D‐MAP effectively induced robust neutralizing antibody titers (Figure 5B), which were slightly lower than those induced by intramuscular (IM) Gardasil but still demonstrated strong immunogenicity, consistent with the ELISA EC50 values. Importantly, the neutralizing antibody titers elicited by 16 V D‐MAP significantly increased following the third vaccination compared to the first, confirming the potent and enhanced induction of neutralizing antibodies.

To examine the generation of plasma cells responsible for producing neutralizing antibodies, we harvested bone marrow where plasma cells typically reside long‐term and quantified antigen‐specific plasma cells by isotype, two weeks after the third immunization. Interestingly, despite the relatively lower levels of antigen‐specific antibodies in the 16 V D‐MAP group compared to the IM Gardasil group, the number of plasma cells secreting immunoglobulin M (IgM), IgG, and IgA was comparable between the two groups (Figure 5C,D). Notably, while IgA was undetectable in the saliva, a considerable number of IgA‐secreting plasma cells were identified in the bone marrow, likely due to the systemic immune response, though the number of IgA spots was lower than that of IgG. This indicates that 16 V D‐MAP effectively induces a robust population of plasma cells, contributing to sustained neutralizing antibody production.

Collectively, these results demonstrate that 16 V D‐MAP induces a strong antibody response with high immunogenicity, including the generation of neutralizing antibodies capable of effectively targeting HPV16, highlighting its potential as a promising vaccine platform for VLP‐based antigen.

### 2.6 Buccal Immunization with 16 V D‐MAP Provides Sterile Protection at Both Oral and Vaginal Mucosal Sites and Induces Buccal Tissue Resident Memory Lymphocytes

**Figure 6:**
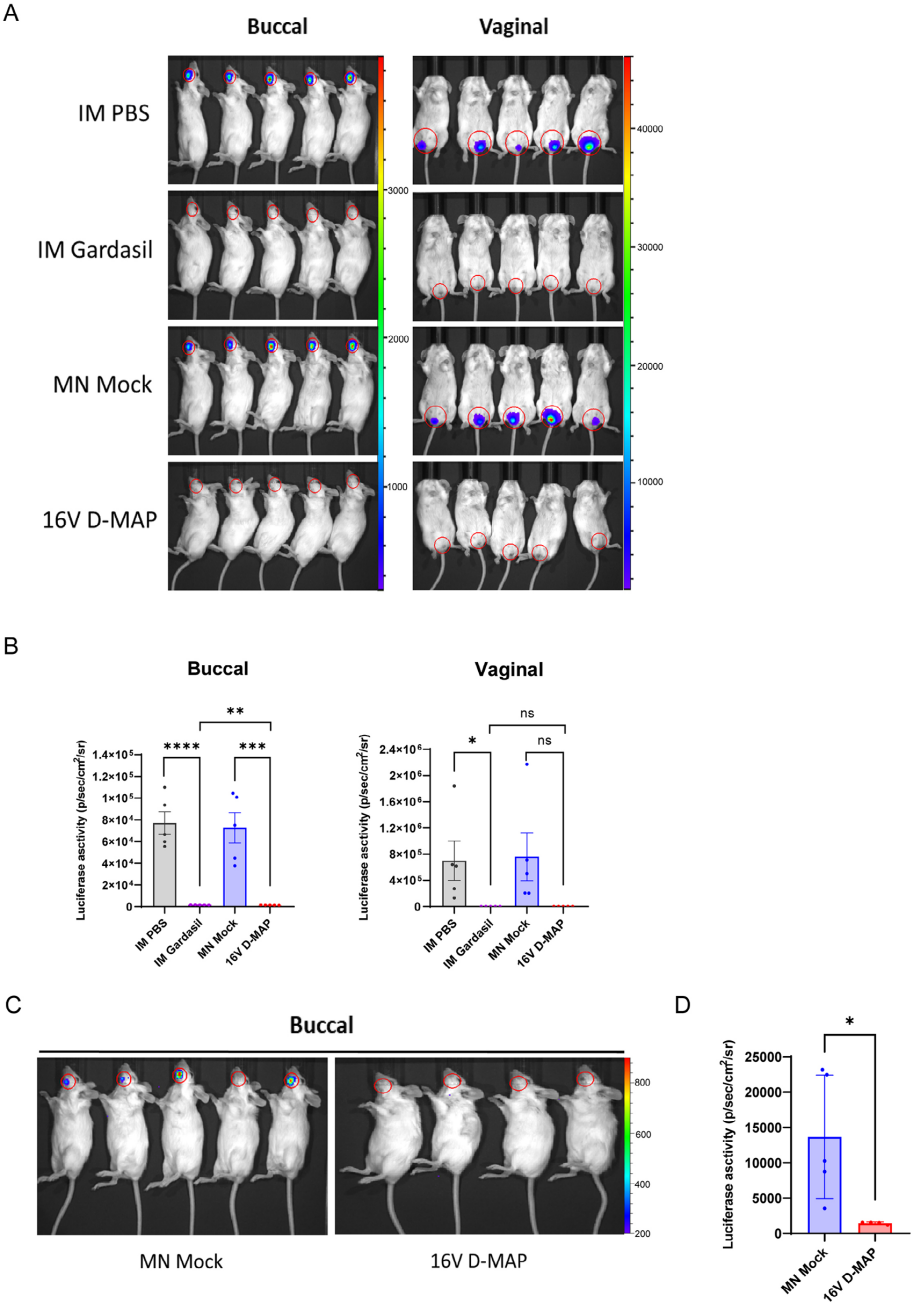
Protection of mice from buccal and genital challenge with HPV16 PsV by IM PBS, IM Gardasil, MN Mock, or 16 V D‐MAP immunization. A) Bioluminescence imaging of buccal and vaginal mucosal areas in vaccinated mice (n = 5 per group) challenged with HPV16 PsV 14 days after the third immunization. Luminescence scales: buccal, 300–3900; vaginal, 1000–46000. B) Quantitative analysis of bioluminescence imaging data from buccal and vaginal areas. C) Bioluminescence imaging of naïve mice passively transferred with antisera and challenged with buccal HPV16 PsV. Luminescence scales: 200–900. D) Quantitative analysis of HPV16 bioluminescence imaging data from buccal areas. Data are presented as means ± SEM. Statistical significance: ns (not significant), *p* > 0.05; *p < 0.05; **p ≤ 0.01; ***p ≤ 0.001; ****p ≤ 0.0001 (unpaired *t*‐test).

Based on the in vitro neutralizing antibody assay, we conducted a mouse challenge study to assess whether 16 V D‐MAP immunization could protect mice from HPV16 PsVs oral infection. Additionally, given reports that robust mucosal vaccination can elicit a broad immune response across multiple mucosal tissues,^[^ ^33^ ^]^ we explored whether buccal immunization with 16 V D‐MAP could provide protection not only against oral infection but also against vaginal infection with HPV16. Following three doses, the 16 V D‐MAP group exhibited strong humoral immune responses, achieving sterile immunity against both oral and vaginal challenges, comparable to IM Gardasil (**Figure** **6**A,B). These findings suggest that buccal administration of 16 V D‐MAP can induce immunity across multiple mucosal tissues, highlighting its potential as a promising vaccine candidate for cervical cancer as well as head and neck cancers.

To determine whether this protection was mediated primarily by neutralizing antibodies rather than cellular immunity, we performed passive serum transfer by administering 20 µL of serum from 16 V D‐MAP immunized mice to naïve mice and tested their protection against oral challenge with HPV16 PsVs. Remarkably, even this small volume of immune serum conferred complete protection against a high dose of HPV16 PsV in the oral challenge (Figure 6C,D). These results suggest that 16 V D‐MAP provides cross‐tissue protection predominantly mediated by antibodies.

These data demonstrate that 16 V D‐MAP provides immune protection across multiple mucosal tissues, further supporting its potential as a highly effective mucosal vaccine platform.

### 2.7 16 V D‐MAP Immunized Mice Show Long‐Lasting Immune Response and Protection

An essential attribute of an effective vaccine is its capacity to induce a high titer of protective antibodies and sustain this response over time, ensuring long‐term immunity against future infections. To determine whether 16 V D‐MAP provides durable protection, we evaluated antigen‐specific antibody titers, neutralizing antibody levels, the persistence of buccal tissue‐resident memory lymphocytes, the presence of antigen‐specific long‐lived plasma cells (LLPCs) in the bone marrow, and, critically, conducted in vivo oral and vaginal challenge experiments in mice six months after the third and final vaccination.

**Figure 7:**
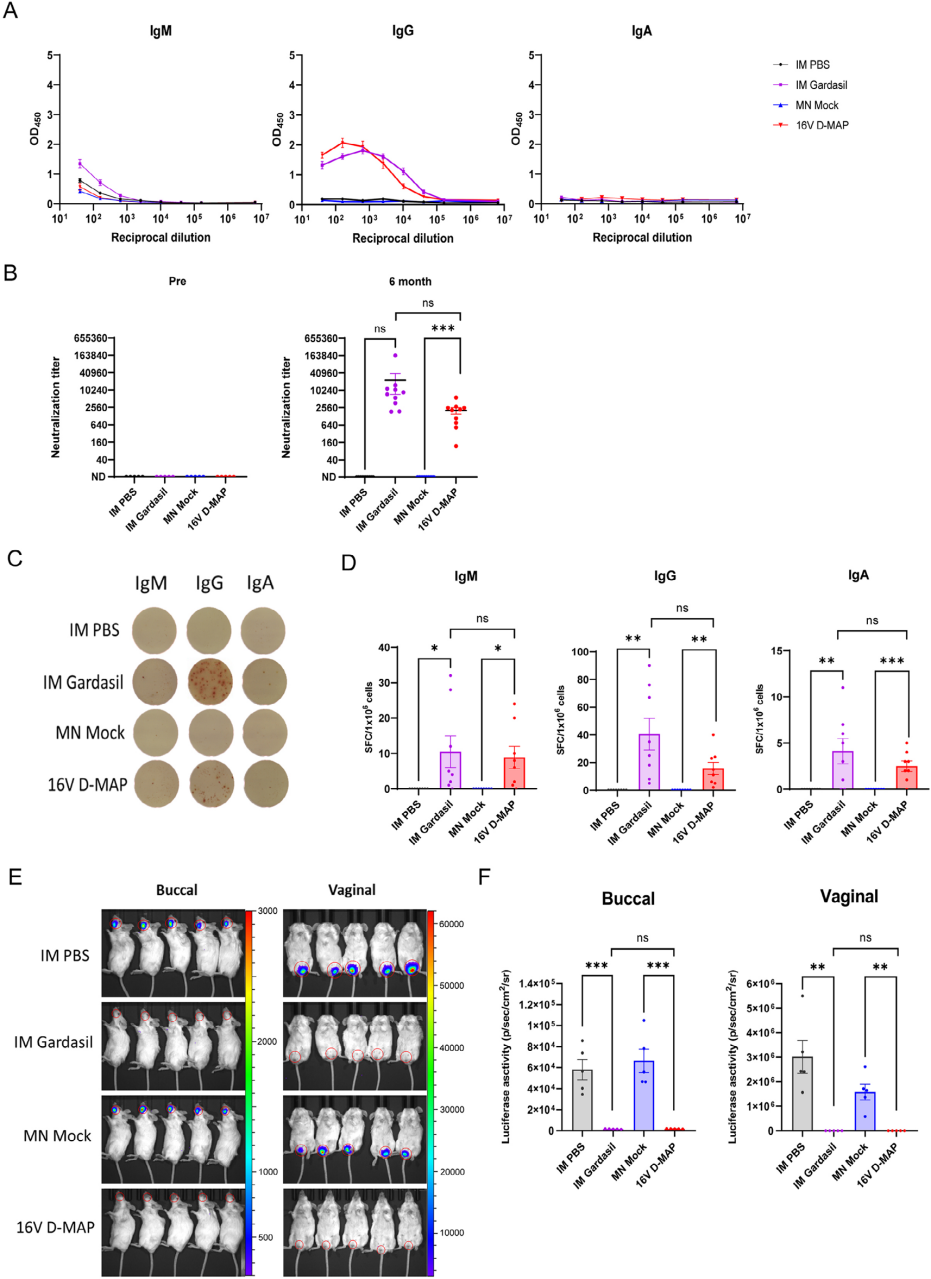
Long‐term immune response and protection against HPV16 VLP 6 months after 3rd immunization. A) Serum levels of HPV16 VLP‐specific IgG, IgM, and IgA in PBS, IM Gardasil, MN Mock, and 16 V D‐MAP groups, detected by ELISA 6 months after the third immunization (n = 10 per group). B) Serum neutralization titers against HPV16 PsV, measured 6 months after the third immunization (n = 10 per group). C) ELISPOT assay detecting HPV16 VLP‐specific IgG‐, IgM‐, and IgA‐secreting plasma cells in BM 6 months after the third immunization (n = 8 per group). D) Quantification of IgG‐, IgM‐, and IgA‐secreting plasma cells based on ELISPOT data. E) Bioluminescence imaging of buccal and vaginal mucosal areas in vaccinated mice (n = 5 per group) challenged with HPV16 PsV 6 months after the third immunization. F) Quantitative analysis of bioluminescence imaging data from buccal and vaginal areas. Data are presented as means ± SEM. Statistical significance: ns (not significant), p > 0.05; ^*^ *p* < 0.05; ^*^^*^ *p* ≤ 0.01; ^***^ *p* ≤ 0.001 (unpaired *t*‐test).

Six months post‐vaccination, 16 V D‐MAP maintained elevated antigen‐specific antibody titers, as reflected by stable EC50 values (**Figure** **7**A). Although neutralizing antibody titers exhibited a gradual decline over this period, the 16 V D‐MAP group retained more than half of its peak neutralizing titer as compared to the early titer measured 2 weeks after the third immunization (Figure 7B). In contrast, the titers in the IM Gardasil group dropped by over 50%. This indicates a slower decay of neutralizing antibodies in the 16 V D‐MAP group compared to IM Gardasil. Additionally, buccal Brm cells and Trm cells, which are believed to play a key role in controlling future infections, were maintained at significantly high levels (Figure S2, Supporting Information). ELISpot analysis further confirmed the continued presence of HPV16 VLP‐specific plasma cells in the bone marrow, which remained detectable across all isotypes at levels comparable to the IM Gardasil group, indicating long‐term antibody secretion (Figure 7C,D). Lastly, the overall immune response was validated through in vivo oral and vaginal challenge experiments. Notably, six months after the final vaccination, 16 V D‐MAP provided complete sterile immunity in both oral and vaginal tissues, demonstrating its robust and sustained protective effects (Figure 7E,F).

Collectively, these findings indicate that 16 V D‐MAP induces a strong immune response that persists for at least six months, fulfilling important criteria for a preventive vaccine. The platform shows substantial potential for long‐term protection against HPV‐related infections, supporting its efficacy as a promising vaccine candidate.

## 3 Conclusion

Microneedles hold great promise as a transformative tool for vaccine delivery, particularly in LMICs where vaccine accessibility faces significant challenges. Despite the effectiveness of HPV vaccines, their widespread distribution is hindered by the high costs associated with cold chain storage and the limited healthcare infrastructure. Utilizing microneedle technology for HPV vaccines offers a promising solution to these challenges. However, since HPV vaccines rely on highly immunogenic VLPs, maintaining the structural integrity of VLPs is essential for preserving their immunogenicity and vaccine efficacy. Dissolving microneedles offer several advantages, yet the manufacturing process can expose VLPs to environmental stressors that may compromise their integrity. In this study, we presented an optimized process that preserves the structural stability of VLPs throughout microneedle fabrication and dissolution. The resulting 16 V D‐MAP successfully induced key immune markers, commonly used as indicators of vaccine‐induced immune responses, providing sterile immunity and protection across multiple mucosal tissues in both buccal and vaginal tissues of murine models. Additionally, 16 V D‐MAP demonstrated the ability to sustain long‐term immune responses, maintaining high antibody titers and neutralizing antibody levels for at least six months post‐vaccination, indicating its potential for both immediate and prolonged protection. The flexibility of microneedle technology also allows for the development of multivalent HPV vaccines, capable of delivering multiple HPV antigens simultaneously. When considering the practical affordability of HPV vaccines for application in LMICs, the initial manufacturing cost of dissolving microneedle array patches (D‐MAP) may exceed conventional intramuscular vaccines (approximately USD 4.50 per dose via global procurement). However, preliminary estimates indicate that competitive pricing (USD 3.5–4.0 per dose) is achievable through large‐scale production and World Health Organization (WHO) prequalification. Moreover, logistical advantages such as simplified administration and reduced cold‐chain dependence could significantly enhance overall cost‐effectiveness and accessibility in resource‐limited settings. Regarding the choice of adjuvant used in this study, cholera toxin A1 subunit (CTA1) served effectively as a proof‐of‐concept mucosal adjuvant for preclinical evaluation. However, given its toxicity concerns for human application, alternative mucosal adjuvants with improved safety profiles, such as nano‐alum, Toll‐like receptor (TLR) agonists, or cyclic diguanylate monophosphate (cyclic‐di‐GMP), will be pursued in future translational studies. Therefore, an HPV vaccine based on 16 V D‐MAP technology could overcome the cost and cold chain storage challenges, offering a practical and scalable solution for vaccine distribution in LMICs. This advancement brings us closer to achieving widespread HPV vaccine coverage in regions where protection against HPV‐related cancers is most urgently needed.

## 4 Experimental Section

### HPVL1 VLP

HPV16 L1 virus‐like particles (VLPs) were prepared by Optipharm (Cheongju, South Korea) and characterized using dynamic light scattering (DLS) and transmission electron microscopy (TEM). The HPV16 L1 VLPs (>90% purity) were suspended in PBS containing 500 mM NaCl and 0.01% Tween 80. The VLP concentration was determined using a Pierce Micro BCA Assay Kit (Thermo Scientific, Rockford, IL, USA) and enzyme‐linked immunosorbent assay (ELISA). DLS measurements were performed with 100 µL of VLP solution, diluted to a final concentration of 20 µL mL^−1^, using a LITESIZER 500 (Anton Paar, Graz, Austria) in plastic disposable microcuvettes. The detailed measurement parameters were as follows: Temperature, 25.0 °C; Equilibration time, 120 s; Measurement angle, 90° Backscatter; Measurement duration, Automatic; Measurement time, 3; Delay between measurements, 30 s; Solvent, PBS. The particle size was calculated from the weighted average of the intensity distribution and was reported as the hydrodynamic diameter (Z‐average) and polydispersity index (PDI). TEM analysis was conducted using an HT7800 (Hitachi, Tokyo, Japan). Samples were prepared by mounting 0.2 mg mL^−1^ VLPs onto 400‐mesh hexagonal copper grids and staining them with 2% (w/v) uranyl acetate.

### Cholera Toxin A1 Subunit

The cholera toxin A1 subunit (CTA1) protein was expressed and purified as previously described (Kim et al.). Briefly, the CTA1 plasmid was transformed into E. coli BL21 (DE3), and the expressed proteins were subsequently purified. The purified proteins were stored at −70 °C until use.

### Fabrication Process of Dissolving Microneedle Array Patch

To manufacture the 16 V D‐MAP, tip and base solutions were prepared separately using micro‐molding technology (Park et al., 2005; Park et al., 2007). The overall fabrication process is illustrated in Figure 1A. A scanning electron microscope (SEM; JSM‐7500F, JEOL, Tokyo, Japan) was used to image the microneedle master mold. The master, fabricated from aluminum sheets, featured 21 pyramid‐shaped tips on a 3 mm circular base (Figure 1B). Each microneedle had a height of 550 µm, a width of 280 µm, a tip‐to‐tip interval of 580 µm, and a base‐to‐base interval of 300 µm. Before preparing the 16 V D‐MAP, a negative replica of the master mold was created using polydimethylsiloxane (PDMS; SYLGARD 184, Dow Corning). Trehalose was included during the manufacturing process to preserve the structural and conformational stability of the solid‐state HPV16 L1 VLP. In the basic formulation (excluding trehalose‐specific studies), the tip solution contained 0.05% (w/v) VLPs, 0.01% (w/v) CTA1, 2.5% (w/v) D‐(+)‐trehalose dihydrate (Sigma Aldrich, St. Louis, MO, USA), and 1.62% (w/v) carboxymethyl cellulose sodium salt (low‐viscosity CMC, Sigma Aldrich). The 16 V D‐MAP base gel was prepared by dissolving 20% (w/v) CMC in distilled water. ≈20 µL of the tip solution was dispensed into the PDMS mold, and the mold cavity, including the tip and the upper part of the base, was filled using a centrifuge set at 5000 × g for 1 h. Subsequently, 30 mg of the microneedle base gel was applied to the mold and centrifuged overnight at 5000 × g to ensure complete drying. The antigen delivery efficiency of microneedles was substantially lower compared to conventional intramuscular injections, with ≈75% efficiency at the tips and ≈5% at the upper portion of the base. Consequently, the tip and upper portion of the base of the 16 V D‐MAP contain a total of 10 µg HPV16 L1 VLP (5 µg each) and 2 µg of CTA1.^[^ ^34^, ^35^ ^]^ Characterization of the 16 V D‐MAP was conducted on lots used for a feasibility study in a mouse model. This included assessments of appearance, antigen content, VLP size and structure, and integrity. VLPs were extracted from the 16 V D‐MAP dissolved in PBS and analyzed. The microneedle patch's appearance was inspected using an optical microscope with FusionOptics (M205C, Leica, Wetzlar, Germany), while the released VLPs were evaluated using ELISA, DLS, TEM, and protein electrophoresis.

### Enzyme‐Linked Immunosorbent Assay

Two types of ELISA were performed to evaluate (1) the antigen content of the 16V D‐MAP using a sandwich ELISA and (2) serum antibody responses using an indirect ELISA.

1. Antigen Quantification in D‐MAP: The antigen content of the 16 V D‐MAP was quantified using a sandwich ELISA. A Maxisorp 96‐well microtiter plate (Thermo Scientific) was coated overnight at 4 °C with a mouse monoclonal antibody specific to HPV16 L1 (CamVir1, Novus Biologicals, Centennial, CO, USA). After coating, the plate was blocked with Invitrogen ELISA Assay Buffer (Thermo Scientific) for 1 h at 37 °C. HPV16 L1 VLP (Optipharm, Cheongju, South Korea) was used as a standard, and dissolved D‐MAP samples were incubated in the wells for 1 h at 37 °C. The antigen was detected using a rabbit polyclonal antibody to HPV16 L1 conjugated with horseradish peroxidase (HRP; Creative Diagnostics, Shirley, NY, USA), which was incubated for 2 h at 37 °C. Following extensive washing, the reaction was developed using 3,3´,5,5´‐tetramethylbenzidine (TMB) substrate, and the reaction was stopped. Absorbance was measured at 450 nm (specific signal) and 620 nm (background signal) using a FLUOstar Omega plate reader (BMG LABTECH, Ortenberg, Germany). A standard curve was generated and analyzed using either a log‐log or 4‐parameter curve fitting method.
2. Serum Antibody Titer: To evaluate vaccine‐induced humoral responses, serum antibody titers were measured by indirect ELISA. A 96‐well EIA/RIA plate (Corning, NY, USA) was coated with HPV16 pseudovirus (PsV) at a concentration of 25 ng/100 µL per well in PBS and incubated overnight at 4 °C. Plates were washed with PBS containing 0.05% Tween‐20 (PBST) and blocked with 1% bovine serum albumin (BSA; Sigma‐Aldrich, St. Louis, MO, USA) in PBS for 2 h at 37 °C. Whole blood was collected from the orbital venous plexus of immunized mice. Serum samples were serially diluted 4‐fold (eight dilution points) in 1% BSA/PBS and incubated for 2 h at 37 °C. After washing, HRP‐conjugated goat anti‐mouse IgG, IgM, and IgA antibodies (SouthernBiotech, Birmingham, AL, USA) were added and incubated overnight at 4 °C. TMB substrate was added to develop color, and the reaction was stopped with 0.5 N HCl. Absorbance was measured at 450 nm using a GloMax® microplate reader (Promega, Madison, WI, USA).

### Protein Electrophoresis

To evaluate the protein composition of the 16 V D‐MAP and assess the integrity of the intact HPV16 L1 monomer, the VLP samples were analyzed using the Agilent 2100 Bioanalyzer system with the Agilent Protein 230 Kit (Agilent, Santa Clara, CA, USA) under reducing conditions. Briefly, denatured samples, prepared under reducing conditions, along with the protein ladder, were loaded into the gel‐dye mix on the loading chip. The chip was then placed in the Agilent 2100 Bioanalyzer, and the analysis was initiated immediately. Protein sizes in the HPV16 L1 VLP samples, ranging from 14 to 230 kDa, were determined. The percentage of intact HPV16 L1 monomer (corresponding to 56 kDa) was calculated as the ratio of the monomer to the total protein content, expressed as a percentage.

### Skin Insertion Test and Dissolution Kinetics of HPV D‐MAP

To confirm the mechanical strength of the 16 V D‐MAP for transdermal application, full‐thickness porcine skin (3 × 3 cm; Cronex, Seoul, South Korea) was secured to a flat surface. The 16 V D‐MAP was manually pressed against the skin using a thumb, applying an approximate force of 10 N for 10 s. Successful skin puncture was verified by staining the skin with trypan blue (Sigma Aldrich). Stained dots on the stratum corneum were observed under an optical microscope, and penetration efficiency was calculated as the ratio of stained puncture holes to the 21 pyramid‐shaped tips in the 16 V D‐MAP. The dissolution kinetics of the 16 V D‐MAP were assessed using a similar skin insertion method. The shape and integrity of the 16 V D‐MAP were evaluated before insertion and at specific time intervals after insertion (10 s, 1, 15, 30, and 60 min). An optical microscope was used to observe the rate and extent of dissolution, comparing the microneedle structure before and after insertion into porcine skin. To further evaluate dissolution kinetics in a mucosal setting more relevant to the intended application, an in vivo insertion test was additionally performed using mouse buccal mucosa. The 16 V D‐MAP was applied onto the buccal mucosa of anesthetized mice and collected at designated intervals (0, 10 s, 1, 5, 10, 20, and 60 min). The recovered patches were dissolved in PBS, and the residual HPV16 L1 antigen content from the microneedle tips was measured by ELISA to calculate antigen delivery efficiency. The morphology and dissolution profile of the 16 V D‐MAP before and after application to mouse buccal mucosa at each time point were observed by SEM (JEOL). These combined results informed the selection of the optimal attachment time for subsequent immunization studies.

### Tissue Imaging

Mouse buccal mucosa was excised and inserted with trypan blue (0.05%)‐ or rhodamine B (0.05%)‐loaded 16 V D‐MAP. Tissues were embedded in optimal cutting temperature (OCT) compound using cryomolds, snap‐frozen, and sectioned at 5 µm thickness using a cryostat (HM525 NX, Thermo Fisher Scientific). Sections were mounted on glass slides and imaged with an EVOS fluorescence microscope (Thermo Fisher Scientific) under bright‐field and fluorescence channels. Merged images were analyzed to evaluate microneedle penetration, cargo retention, and spatial distribution in the tissue.

### Stability Test

Accelerated stability testing was conducted at 40 ± 2 °C with 75 ± 5% relative humidity (RH), while long‐term stability testing was performed at 25 ± 2 °C with 60 ± 5% RH and at 5 ± 3 °C. In the accelerated stability test, the antigen content of the 16 V D‐MAP was measured on days 0 and 7. For the long‐term stability test, measurements were taken at 0, 1, 2, 3, and 6 months. The stability data collected at each time point were analyzed using linear regression to assess changes in antigenicity over time (per day or per month). The slope of the regression line and the corresponding 95% confidence interval (CI) were calculated to quantify the rate of antigen degradation.

### Animal

All mice were maintained under specific pathogen‐free conditions. Animal experiments were approved by the Institutional Animal Care and Use Committee (IACUC) of Yonsei University College of Medicine (approval number: IACUC‐2021‐0188) and conducted in accordance with the approved ethical guidelines. Female BALB/c mice (6 weeks old; Orient Bio) were purchased and acclimated for one week prior to experimentation.

### In Vivo Vaccine Immunization

Mice immunized with VLP via microneedle received 10 µg of the 16 V D‐MAP or MN Mock, both designed and manufactured by QuadMedicine (Seongnam, South Korea). Immunizations were administered either as a single dose or as three doses at two‐week intervals. The PBS control group received intramuscular injections of PBS into the right quadriceps femoris muscle, while the IM Gardasil group received Gardasil‐4 at a dosage equivalent to one‐tenth of the human dose administered into the same muscle site as the PBS control. For the 16 V D‐MAP and MN Mock groups, microneedle patches were applied onto the left buccal mucosa of anesthetized mice for 20 min to ensure complete dissolution and antigen delivery.

### Tissue Resident Leukocyte Isolation

Mice were pretreated with 10 µg mL^−1^ anti‐mouse CD45 antibody (Clone 30‐F11, BioLegend) via intravenous injection and incubated for 5 min. Afterward, the mice were euthanized in CO₂ chambers, and the oral mucosa tissues were harvested. The harvested tissues were finely chopped and subjected to enzymatic digestion. Tissues were first treated with Liberase Dispase Low (DL) (0.5 mg mL^−1^) (Roche) for 20 min, followed by Liberase Thermolysin Low (TL) (0.25 mg mL^−1^) (Roche) for another 20 min at 37 °C under continuous rotation. The protease reaction was stopped by adding 1 mM EDTA, and the digested tissues were filtered through a 70‐µm cell strainer. Cells were collected, washed with PBS, and centrifuged at 300 × g for 8 min. FACS staining was performed following the same protocol as for flow cytometry.

### Flow Cytometry

Mice were euthanized in CO₂ chambers prior to organ collection, and cervical and draining lymph nodes were surgically harvested and placed in FACS buffer (0.5% BSA in PBS). Single‐cell suspensions were prepared by mechanical dissociation, and red blood cells were lysed using ACK lysis buffer (Biosesang, South Korea). The resulting cells were resuspended in FACS buffer, and Fc receptors were blocked with purified anti‐mouse CD16/32 antibody (BioLegend) for 10 min. Cells were subsequently stained on ice for 20 min with appropriately diluted antibodies, including LIVE/DEAD Fixable Near‐IR Dead Cell Stain Kit (Invitrogen); CD19 (6D5), B220 (RA3‐6B2), CXCR5 (L138D7), PD‐1 (29F.1A12), CD38 (90), IgM (RMM‐1), CD103 (2E7), IgD (11‐26c.2a), CD3 (17A2), and IgG (Poly4053) from BioLegend; CD95 (Jo2), GL‐7 (GL7), CD138 (281‐2), and CD69 (H1.2F3) from BD Biosciences; and IgA from Southern Biotech. Following staining, cells were washed twice with FACS buffer and analyzed using a Celesta or LSR Fortessa flow cytometer (BD Biosciences). Data were processed and analyzed using FlowJo software.

### Immunofluorescence Imaging

Cervical lymph nodes were harvested in FACS buffer, snap‐frozen in O.C.T. compound (Sakura Finetek USA) within cryo‐molds, and stored at −80 °C. Tissue sections, 5 µm thick, were prepared using a cryostat (Thermo Scientific). Sections were dehydrated with acetone chilled to −20 °C for 2 min, and residual O.C.T. compound was removed using PBST (0.05% Tween 20 in 1X PBS). Blocking was performed with 3% BSA and 10% goat serum (ThermoFisher Scientific) in PBS for 30 min at room temperature. The sections were then stained with anti‐mouse IgD, anti‐mouse TCR‐beta, and anti‐mouse GL‐7 antibodies (BioLegend) for 2 h at room temperature. After staining, sections were mounted using ProLong Gold Antifade Mountant (Invitrogen). Images were captured using the THUNDER Imaging System (Leica), and raw data were processed using Fiji/ImageJ software.

### Neutralization Assay

The PsV‐based neutralization assay was performed as previously described, with minor modifications.^[^ ^36^ ^]^ HEK 293TT cells were seeded at a density of 3 × 10⁴ cells per well in a 96‐well plate and incubated for 6 h. Serum samples were serially diluted fourfold in culture medium, and HPV16 PsV was added to the diluted serum mixture. The mixture was incubated at 4 °C for 1 h on a rocking shaker and then added to the HEK 293TT cell‐seeded plate. Plates were incubated for 72 h at 37 °C. After incubation, 100 µL of culture medium was removed from each well, and 100 µL of ONE‐Glo luciferase assay reagent (Promega) was added. The plate was incubated on a rocking shaker for 3 min to ensure thorough mixing. The contents of each well were resuspended and transferred to a black 96‐well plate. Luminescence was measured using a GloMax microplate reader (Promega) after transferring the supernatant to a black plate.

### Pseudovirus (PsV) Production

The HPV16 L1‐L2 plasmid was co‐transfected with a plasmid expressing the luciferase reporter gene into HEK 293TT cells using Turbofect transfection reagent as previously described.^[^ ^36^ ^]^ Cells were incubated for 48 h at 37 °C, harvested, washed with DPBS‐Mg (DPBS supplemented with 9.5 mM MgCl₂), and lysed in an equal volume of lysis buffer (DPBS supplemented with 0.5% w/v Brij‐58 and 0.2% v/v RNase cocktail). To facilitate PsV maturation, the cell lysates were incubated at 37 °C for 24 h. For PsV extraction, lysates were centrifuged at 10,000 × g for 10 min, and the clarified supernatants were loaded onto a 12 mL Optiprep step gradient (39%, 33%, 27% w/v) and centrifuged at 280,000 × g for 16 h at 16 °C using an SW41 rotor. Following centrifugation, 1 mL fractions were collected from the top layer, and the fraction with the highest infectivity was selected for subsequent experiments.

### Passive Sera Transfer and Buccal/Vaginal Challenge with HPV Pseudovirus

Female BALB/c mice were injected intraperitoneally with 3 mg of Depo‐Provera. Three days later, a total volume of 100 µL, consisting of 20 µL pooled mouse sera and 80 µL PBS, was administered via intravenous injection. Sera were collected from five mice previously vaccinated with MN Mock or 16 V D‐MAP as described above. After 24 h, mice were vaginally challenged with 10 µL of HPV16 PsV mixed with 10 µL of 3% carboxymethyl cellulose (CMC). A cytobrush was inserted into the vaginal vault and rotated both counter‐clockwise and clockwise 15 times while the mice were anesthetized. In addition, mice were buccally challenged with 10 µL of HPV16 PsV mixed with 10 µL of 3% CMC. The buccal mucosa was scarified using a tattoo needle, with 10 repeated scratches, while the mice were anesthetized. Three days after HPV16 PsV delivery, mice were anesthetized, and 20 µL of luciferin (7.8 mg mL^−1^) was deposited in the vaginal vault and buccal mucosa. Luciferase signals were acquired for 10 min using a Xenogen IVIS 100 imaging system, and data were analyzed using Living Image 2.5 software.

### Statistical Analysis

Quantitative results were expressed as mean values with corresponding standard errors of the mean (± SEM). For comparisons between two independent groups, statistical significance was assessed using an unpaired, two‐tailed Student's *t*‐test. In cases involving more than two groups, a one‐way analysis of variance (ANOVA) followed by Tukey's multiple comparisons test was employed. Statistical significance was defined as *P* < 0.05. Levels of significance were indicated as follows: *P* < 0.05; *P* ≤ 0.01; *P* ≤ 0.001; *P* ≤ 0.0001; with “ns” denoting not significant. All statistical analyses were conducted using GraphPad Prism version 10 (GraphPad Software, San Diego, CA, USA).

## Conflict of Interest

The authors declare no conflict of interest.

## Supporting information



Supporting Information

## Data Availability

The data that support the findings of this study are available in the supplementary material of this article.
