# Supplementary material for: Dissolving Microneedle for Maintaining the Integrity of HPV Virus‐Like Particles Enabling Durable Sterile Protection Across Various Mucosal Tissues
Source: Adv Healthc Mater. 2025 Jul 9;14(24):2500963. doi: 10.1002/adhm.202500963 (PMC12447025; doi:10.1002/adhm.202500963)
Supplement: Supplementary file 1 — Supporting Information [file ADHM-14-0-s001.docx]

Dissolving microneedle for maintaining the integrity of HPV virus-like particles enabling durable sterile protection across various mucosal tissues

*Hyemi Kim^†^, Inhyuk Hwang^†^, Juhee Seo^†^, Chaiwon Kim, In-Jeong Choi, Miran Kang, Min-Seok Rha, Youngjae Hong, Jooyoung Kim, Seung-Ki Baek, Jung-Hwan Park*, Hyung-Ju Cho* and Kihyuck Kwak**

H. Kim, I. Hwang, C. Kim, Y. Hong, K. Kwak

Department of Microbiology and Immunology

Yonsei University College of Medicine

50–1 Yonsei-ro, Seodaemun-gu, Seoul 03722, Republic of Korea

E-mail: KIHYUCKKWAK@yuhs.ac

I. Hwang, C. Kim, Y. Hong, H.-J. Cho, K. Kwak

Brain Korea 21 Project for Medical Science

Yonsei University College of Medicine

50–1 Yonsei-ro, Seodaemun-gu, Seoul 03722, Republic of Korea

J. Seo, M. Kang, M.-S. Rha, H.-J. Cho

Department of Otorhinolaryngology

Yonsei University College of Medicine

50–1 Yonsei-ro, Seodaemun-gu, Seoul 03722, Republic of Korea

M.-S. Rha, H.-J. Cho

The Airway Mucus Institute

Yonsei University College of Medicine

50–1 Yonsei-ro, Seodaemun-gu, Seoul 03722, Republic of Korea

I.-J. Choi, J. Kim, S.-K. Baek

QuadMedicine R&D Centre

QuadMedicine, Inc.,

sagimakgol-ro, 45beon-gil, jungwon-gu, seongnam-si, Gyeonggi-do 13209, Republic of Korea

J.-H. Park

Department of BioNano Technology

Gachon University

**1342, Seongnam-daero, Sujeong-gu, Seongnam-si, Gyeonggi-do 13120, Republic of Korea**

Keywords: dissolving microneedle, buccal vaccination, mucosal immunity, human papilloma virus, neutralizing antibodies, long-term immune response

**Supporting information**

*Hyemi Kim^†^, Inhyuk Hwang^†^, Juhee Seo^†^, Chaiwon Kim, In-Jeong Choi, Miran Kang, Min-Seok Rha, Youngjae Hong, Jooyoung Kim, Seung-Ki Baek, Jung-Hwan Park*, Hyung-Ju Cho* and Kihyuck Kwak**

**
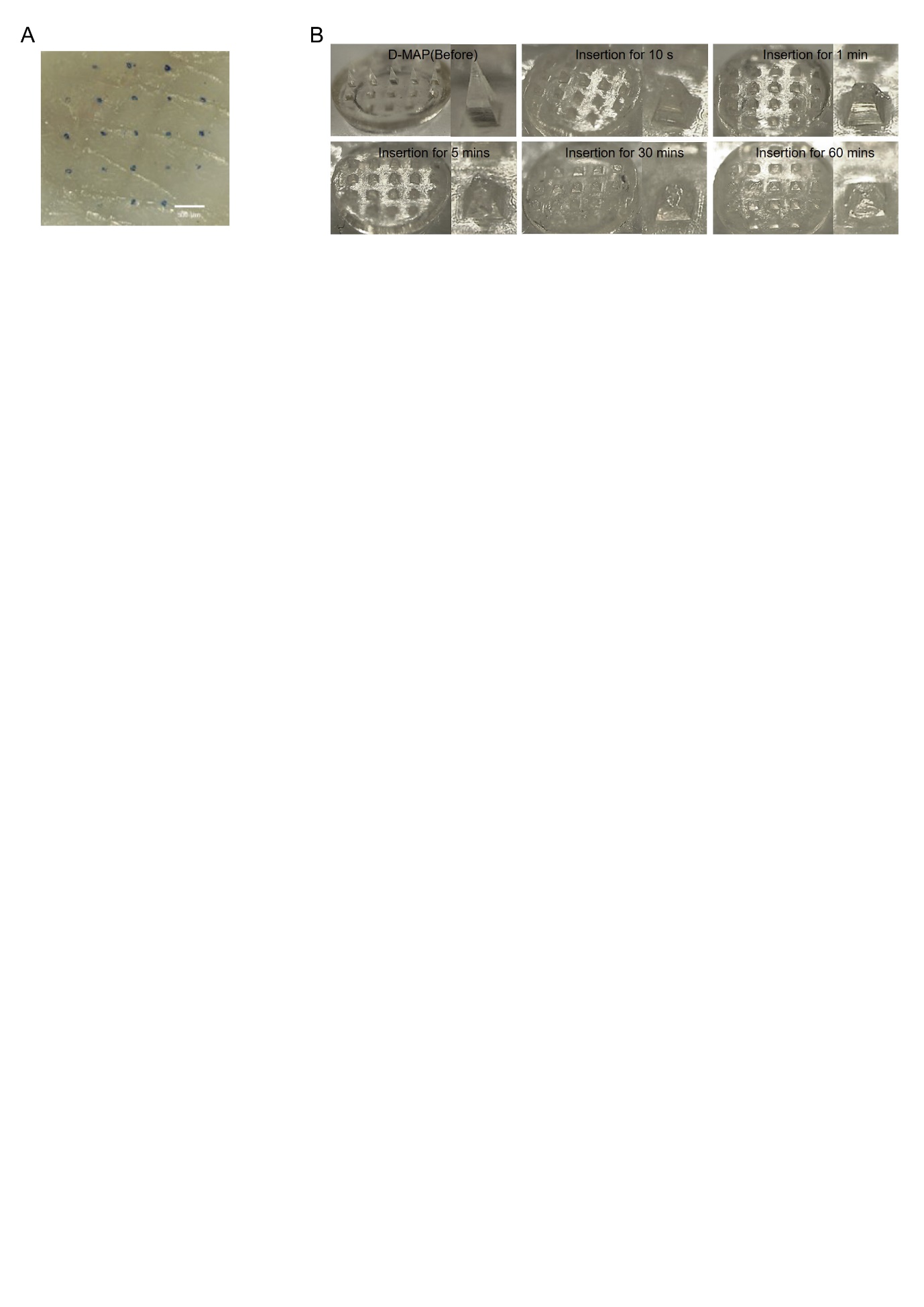
**

**Figure S1.** Insertion efficiency and dissolution behavior of HPV D-MAP in porcine skin. (A) Penetration test results of HPV D-MAP on porcine skin. (B) Dissolution kinetics of HPV D-MAP before and after insertion into porcine skin at intervals of 10 seconds, 1, 15, 30, and 60 minutes.

**
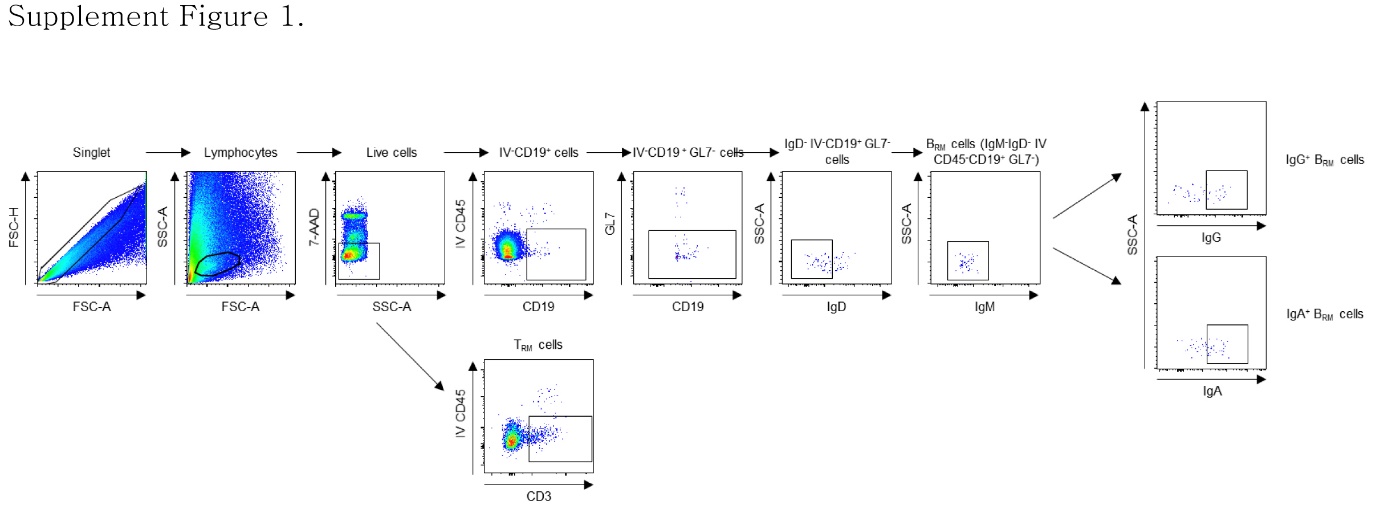
**

**Figure S2.** FACS gating strategy for the analysis of buccal mucosal Brm, Trm, and plasma cells.

**
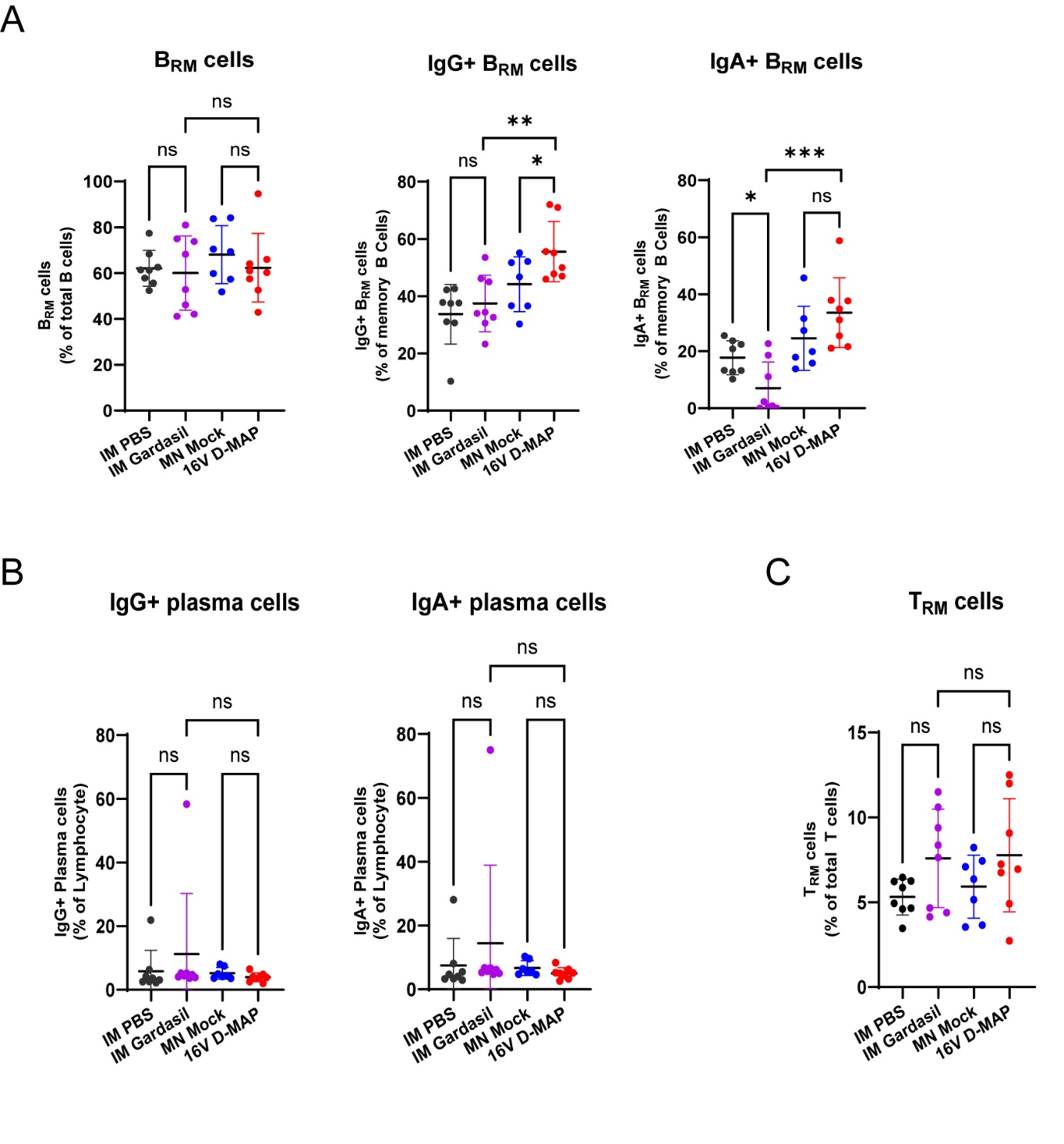
 Figure S3.** Long-term immune memory after 16V D-MAP immunization. (A-C) Statistical analysis of Brm, Trm, and plasma cells in the buccal tissue of mice 6 months after the third immunization with IM PBS, IM Gardasil, MN Mock, or 16V D-MAP (n = 8 per group). Data are presented as means ± SEM. ns (not significant), p > 0.05; *P < 0.05; **P ≤ 0.01; **** P ≤ 0.0001 (unpaired t test).


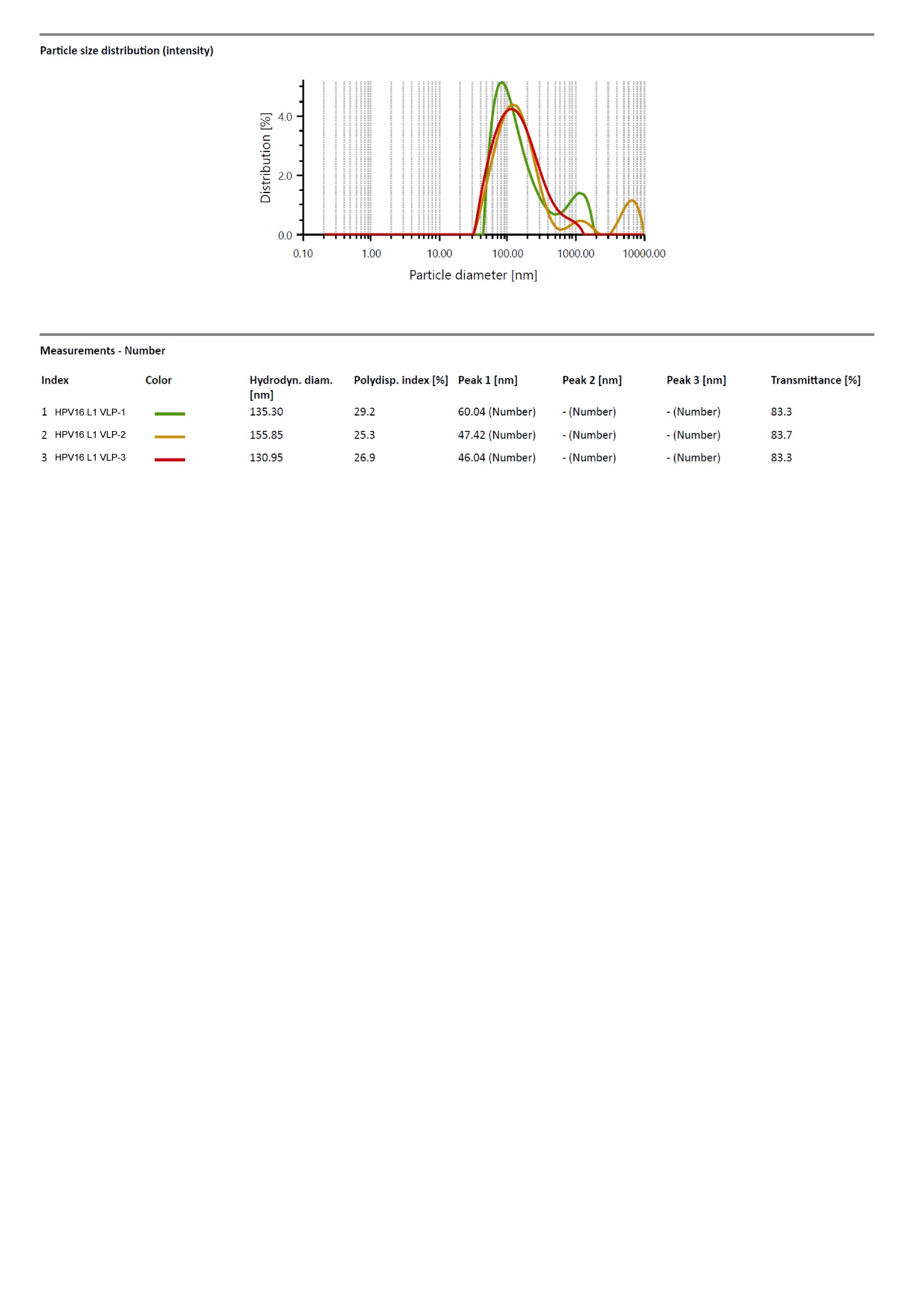


**Table S1.** Raw DLS data of HPV16 L1 VLPs, showing hydrodynamic diameter, polydispersity index, peak sizes, and transmittance from three measurements using Litesizer 500 (Figure 1G).


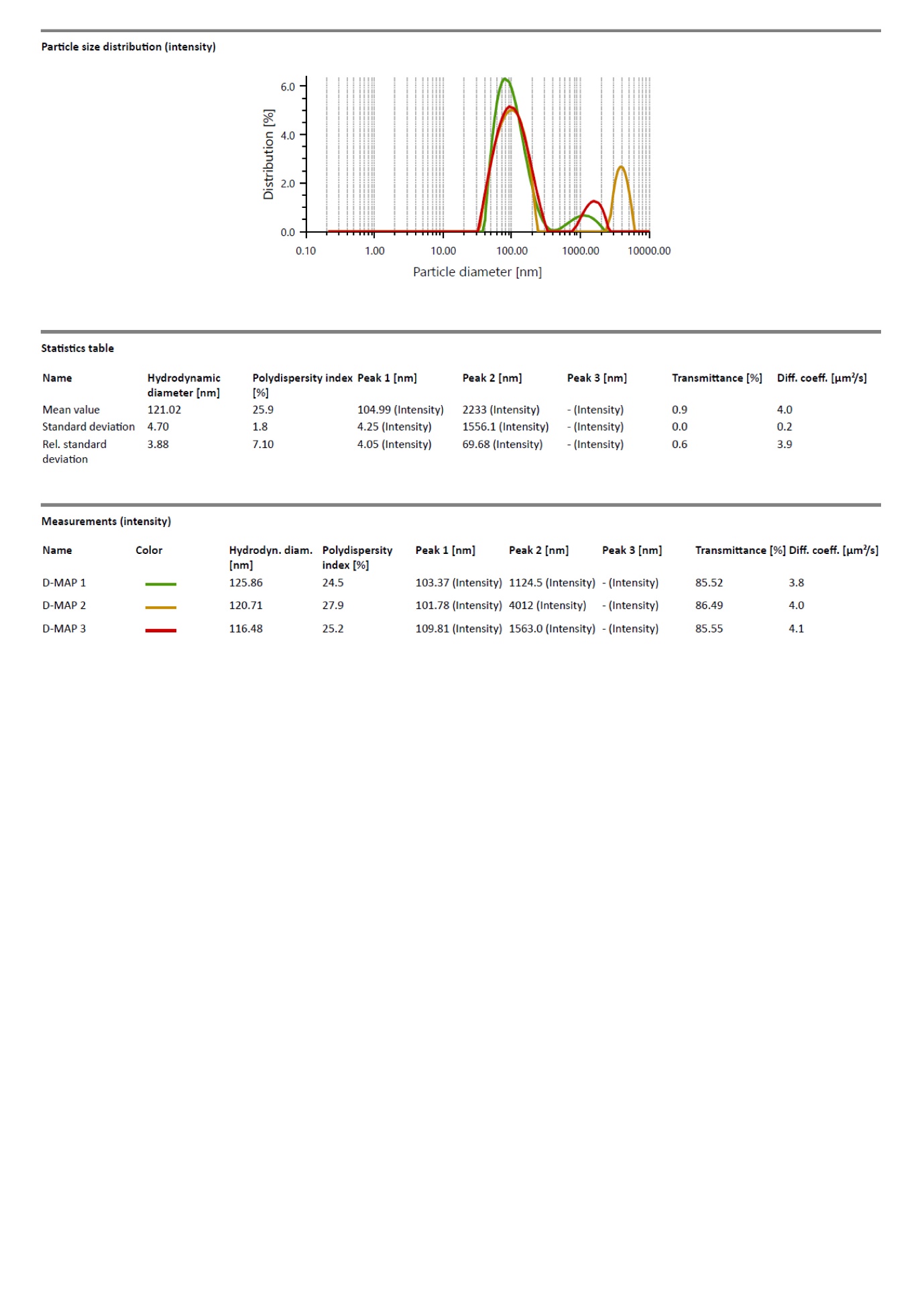


**Table S2.** Raw DLS data of D-MAP particles, corresponding to Day 0 1:50 of VLP to trehalose formulation (Figure 1G and 2D).


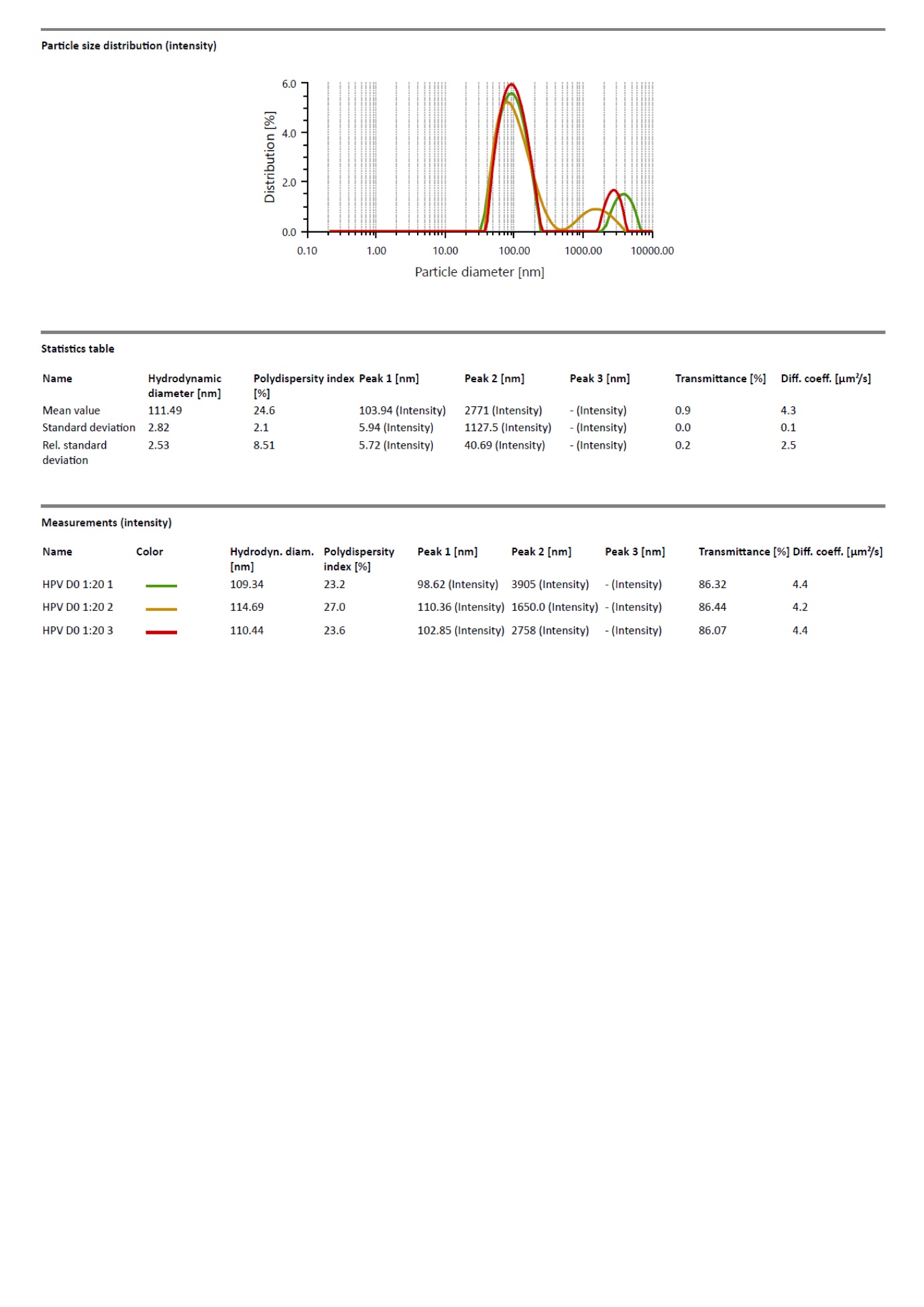


**Table S3.** Raw DLS data of D-MAP particles, corresponding to Day 0 1:20 of VLP to trehalose formulation (Figure 2D).


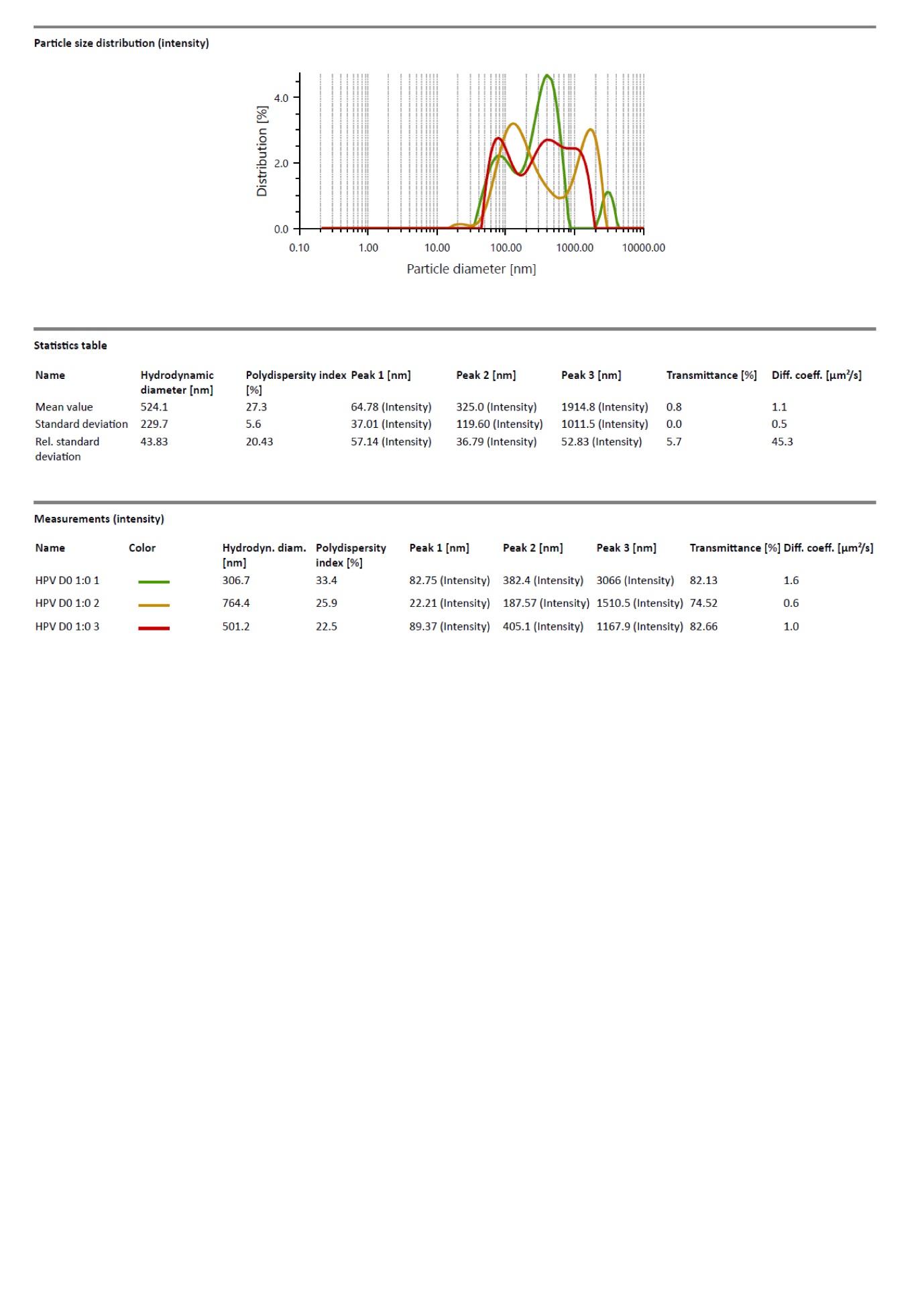


**Table S4.** Raw DLS data of D-MAP particles, corresponding to Day 0 1:0 of VLP to trehalose formulation (Figure 2D).


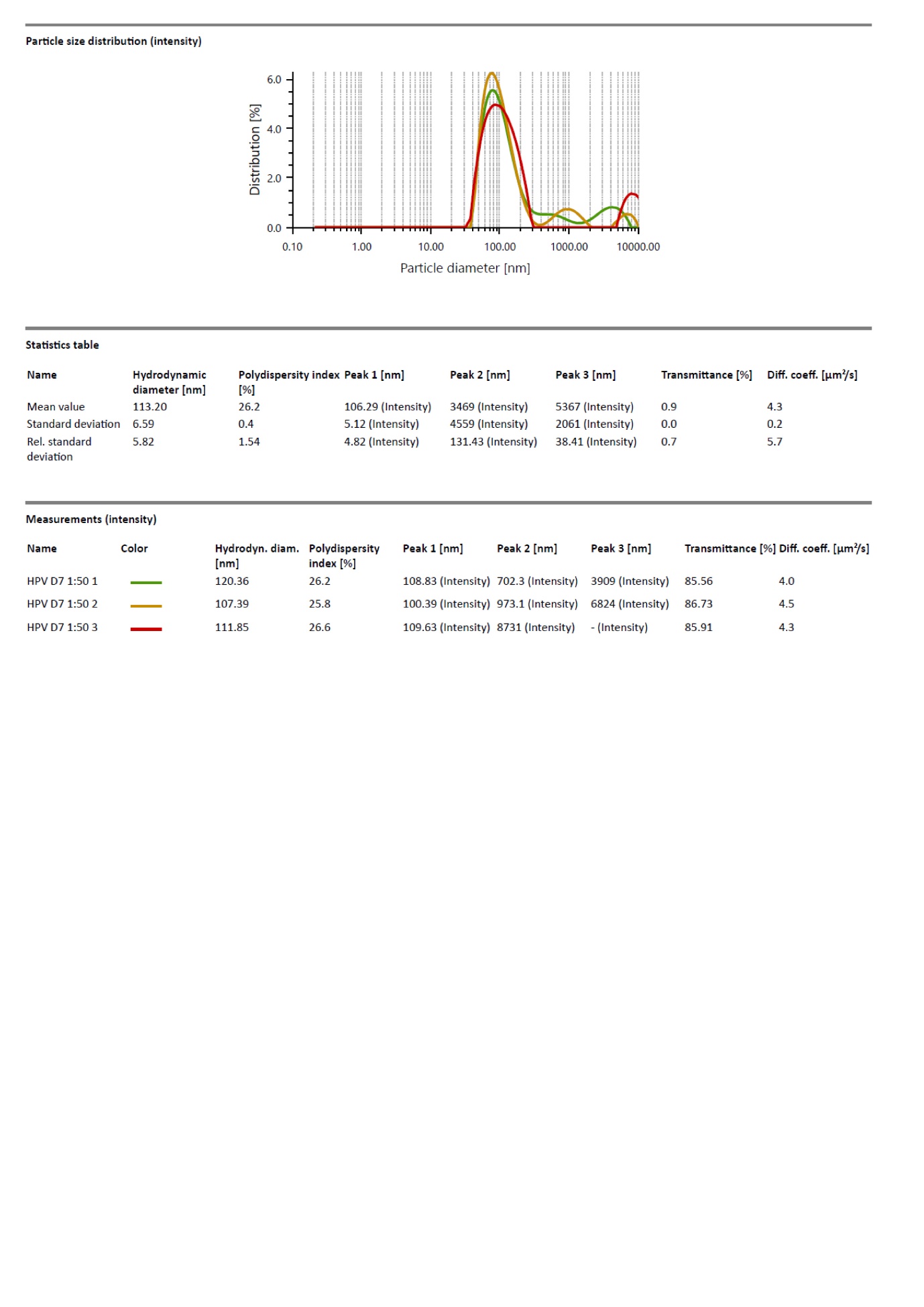


**Table S5.** Raw DLS data of D-MAP particles, corresponding to Day 7 1:50 of VLP to trehalose formulation (Figure 2E).


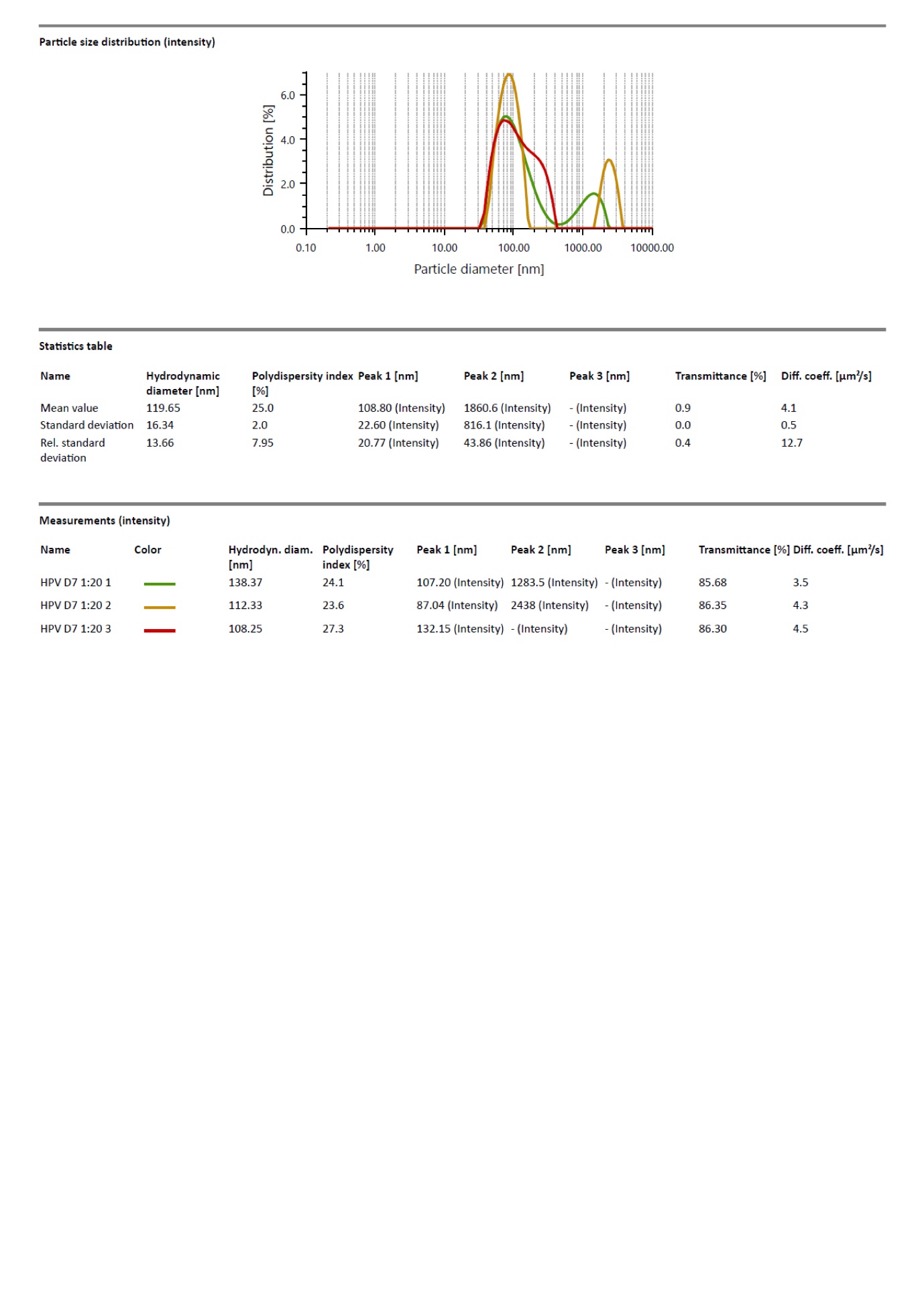


**Table S6.** Raw DLS data of D-MAP particles, corresponding to Day 7 1:20 of VLP to trehalose formulation (Figure 2E).


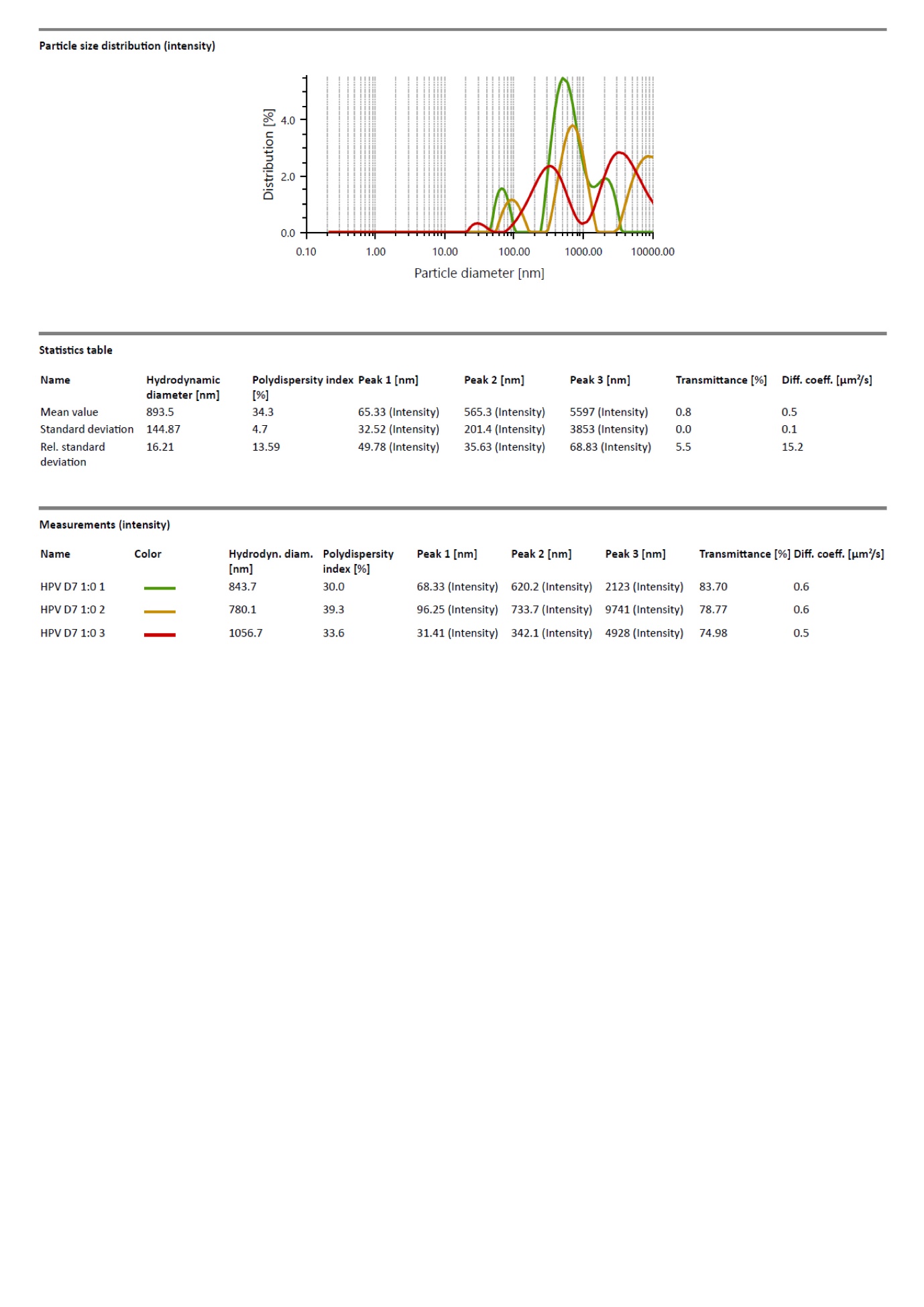


**Table S7.** Raw DLS data of D-MAP particles, corresponding to Day 7 1:0 of VLP to trehalose formulation (Figure 2E).
